# Molecular characterization of colorectal cancer liver metastases with pure desmoplastic versus non-pure desmoplastic histopathological growth patterns using a targeted gene expression panel

**DOI:** 10.1007/s10585-026-10433-x

**Published:** 2026-10-12

**Authors:** Felix Keil, Andreas Lösch, Marco Mühlbauer, Tobias Pukrop, Raquel Blazquez, Hans Jürgen Schlitt, Karolina Müller, Maria Steiger, Yanren Linda Hu, Julia Maurer, Cathleen Brese, Michael Gerken, Matthias Evert, Daniela Hirsch, Rainer Spang, Stefan M. Brunner, Katja Evert

**Affiliations:** 1 Institute of Pathology, University of Regensburg, 93053 Regensburg, Germany; 2 Department of Statistical Bioinformatics, University of Regensburg, 93053 Regensburg, Germany; 3 Center for Translational Oncology, University Hospital Regensburg, 93053 Regensburg, Germany; 4 Bavarian Cancer Research Center (BZKF), 93053 Regensburg, Germany; 5 Department of Internal Medicine III, Hematology and Oncology, University Hospital Regensburg, 93053 Regensburg, Germany; 6 Fraunhofer Institute for Toxicology and Experimental Medicine ITEM-R, 93053 Regensburg, Germany; 7 Department of Surgery, University Hospital Regensburg, 93053 Regensburg, Germany; 8 Center for Clinical Studies, University Hospital Regensburg, 93053 Regensburg, Germany; 9 Health Data Technologies GmbH, 74172 Neckersulm, Germany; 10 University Cancer Center Regensburg (UCC-R), 93053 Regensburg, Germany; 11 Tumor Center Regensburg, Center of Quality Management and Health Services Research, University of Regensburg, 93053 Regensburg, Germany

**Keywords:** Colorectal cancer liver metastasis (CRCLM), Histopathological growth pattern (HGP), Macrometastasis/organ parenchyma interface (MMPI), Gene expression analysis

## Abstract

The histological growth pattern (HGP) at the interface of colorectal adenocarcinoma liver metastases (CRCLM) and liver parenchyma provides important prognostic information. This study aimed to identify transcriptomic signatures underlying the predominant HGPs in CRCLM. We retrospectively evaluated the HGP of 495 CRCLM of 232 patients with metastasectomy at the University Hospital Regensburg between 2003 and 2016. A representative subset of 106 metastases from 53 patients was analyzed for RNA expression at the metastasis–liver interface using the NanoString nCounter® PanCancer Progression Panel. The most common HGPs in the NanoString subcohort were the infiltrative replacement (rHGP, 47.2%) and the non-infiltrative desmoplastic type (dHGP, 46.3%). Unsupervised clustering of CRCLM gene expression profiles identified four gene clusters, two significantly enriched for pure dHGP or pure rHGP. Differential expression analysis between pure dHGP and all other HGPs (i.e., non-pure dHGP) identified 55 significantly differentially expressed genes. Gene set enrichment analysis corroborated previously reported signals (extracellular matrix organization, stromal/endothelial gene sets, and epithelial-mesenchymal transition enrichment in pure dHGP; glucose metabolism in non-pure dHGP) and additionally revealed α6β4 integrin signaling as feature of non-pure dHGP. Patient identity, rather than HGP, was the dominant driver of gene expression variance, though *AGR2* was consistently and significantly overexpressed in non-pure dHGP across different analytical strategies. These findings corroborate known transcriptomic differences between HGPs, identify α6β4 integrin signaling as an additional candidate pathway enriched in non-pure dHGP CRCLM, and underscore the substantial contribution of patient-specific factors to gene expression variability in CRCLM outweighing HGP.

**Supplementary Information:**

The online version contains supplementary material available at https://doi.org/10.1007/s10585-026-10433-x.

## Introduction

The interface between a macrometastasis and the adjacent organ parenchyma is not just a simple boundary where malignant cells meet healthy organ tissue. It is a heterogeneous interface where interactions between tumor cells, the metastatic microenvironment and healthy parenchymal cells take place. This area has been summarized as the macrometastasis/organ parenchyma interface (MMPI) [1]. The MMPI has been particularly well studied in colorectal cancer liver metastases (CRCLM), where a consensus guideline for determining the MMPI_liver_ (the so-called histopathological growth pattern, HGP) has been published [2, 3]. At least one in four patients with colorectal cancer will develop liver metastases [4, 5], either within three months of the primary diagnosis (synchronous metastases, 71.4%) or later during the course of the disease (metachronous metastases, 28.5%) [4]. Complete surgical resection results in a mean 5-year overall survival of 30% [6] and is now firmly established as one of several treatment options for patients with CRCLM. Recently, the multicenter, open-label, randomized TransMet trial even performed liver-transplantation plus chemotherapy in patients with persistent stable disease of unresectable CRCLM with promising results (5-year overall survival for liver-transplantation plus chemotherapy 57% vs. 13% for chemotherapy alone, *p *< 0.001) [7].

Clinical criteria have been developed to select patients for hepatectomy who will benefit from surgical resection of CRCLM [8]. However, patients with poor prognostic clinical factors—such as a node-positive colorectal primary, more than one liver metastasis, metastases in both liver lobes, a tumor size greater than 5 cm, a disease-free interval of less than 12 months, or synchronous metastatic disease–may prove to be long-term survivors after metastasectomy. Hence, resection should not generally be withheld from these patients [9]. In addition to clinical factors, the HGP of the CRCLM has been shown to have a significant impact on overall survival [2, 3, 10, 11]. Patients with liver metastases that are completely surrounded by a desmoplastic (pseudo)capsule (desmoplastic HGP, dHGP) survive significantly longer than those with metastases in which cancer cells replace hepatocytes and other liver parenchymal cells (replacement HGP, rHGP) or those with a mixed type of MMPI [3, 10, 11].

To investigate the molecular features associated with distinct HGPs, previous studies have compared dHGP and rHGP CRCLM using either bulk transcriptomics [12, 13] or spatially resolved and single-cell transcriptomic approaches [11]. Across these studies, dHGP was consistently associated with upregulation of genes involved in inflammatory responses and immune cell recruitment [11–13], angiogenesis [11–13], and epithelial-to-mesenchymal transition (EMT) [12, 13]. In contrast, rHGP exhibited consistent upregulation of genes related to cell proliferation, tumor aggressiveness, and glucose metabolism [11–13].

In addition to comparisons between HGP subtypes, other studies have focused on molecular differences within the desmoplastic rim itself, particularly between the inner rim at the tumor interface and the outer rim adjacent to the liver parenchyma. These region-specific analyses identified evidence of tumor-driven extracellular matrix remodeling in the inner rim, whereas the outer rim displayed molecular features consistent with a benign fibrotic response of the surrounding liver tissue [14, 15].

With the present study, we aim to contribute to the molecular characterization of HGPs in CRCLM through three methodological choices that, to our knowledge, have been underexplored in this context: (1) we assess gene expression using a commercially available targeted panel, allowing us to evaluate which established, and which potentially novel, gene expression signals may represent translational candidates; (2) we specifically contrast pure dHGP against other HGPs, reflecting its distinct clinical relevance; and (3) we include a comparatively high number of samples per patient, allowing us to better assess the contribution of patient-specific factors to overall gene expression variability in this context.

## Materials and methods

### Experimental design

**Fig. 1:**
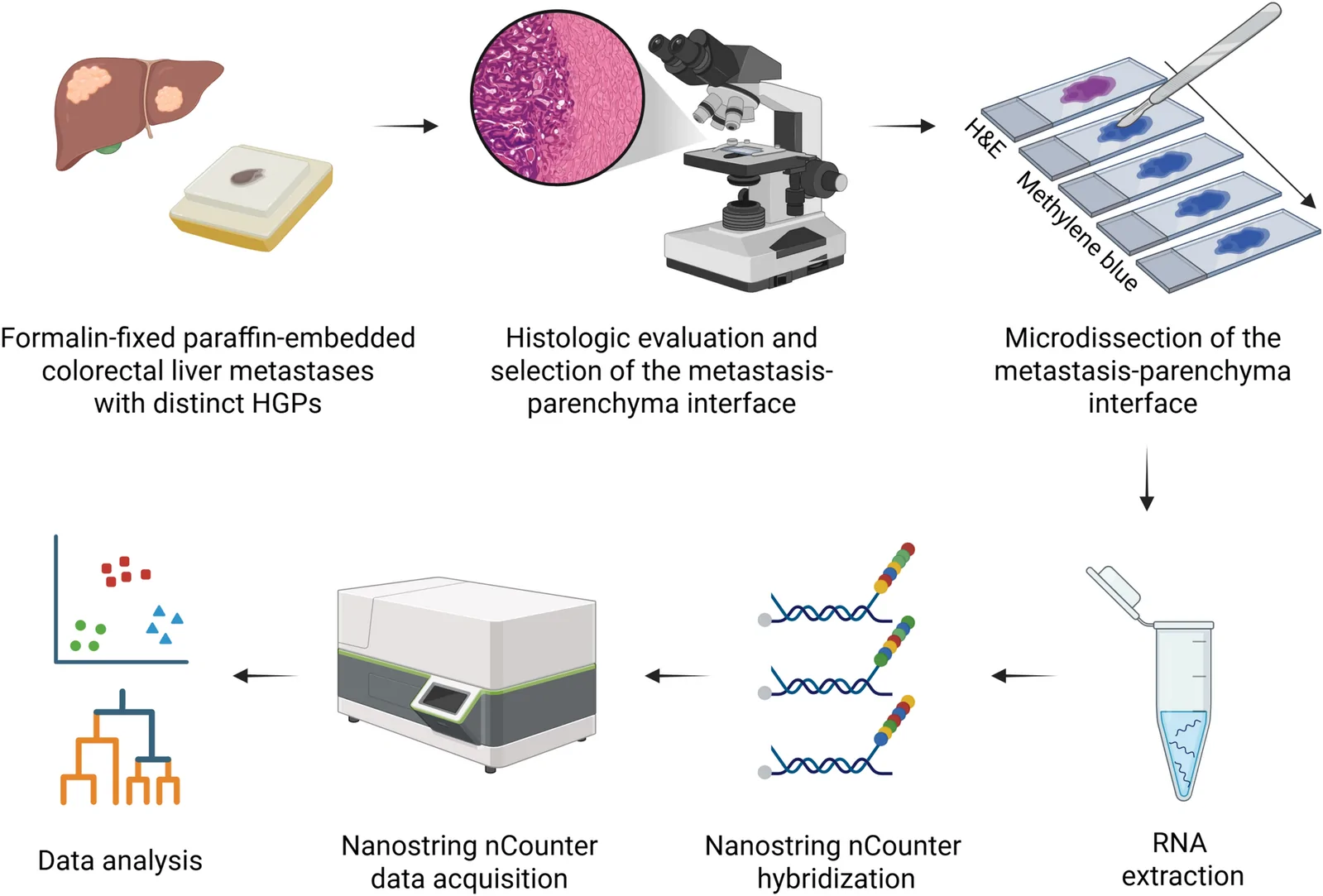
This graphical summary illustrates the overall workflow, from histomorphological HGP assessment through NanoString-based gene expression profiling to downstream data analysis

We retrospectively evaluated a previously established cohort of patients with CRCLM [16] for HGP. A representative subset of these CRCLM was selected based on amount of available tissue and to reflect the relative distribution of HGPs within the cohort. This subset was subjected to gene expression analysis to identify differentially expressed genes and gene sets between HGPs. Associations between HGP and survival and gene expression data and survival were analyzed to explore the prognostic potential of both HGP and differential gene expression data. The workflow underlying this study is shown in Fig. 1.

### Patient cohort

A retrospective cohort of patients who underwent surgical resection of CRCLM at the University Hospital Regensburg, some of whom had in part already been the basis of a previous study [16], was provided by SMB and HJS. Formalin-fixed, paraffin-embedded (FFPE) tissue from these liver metastases as well as from the primary tumors, if available, was obtained from the archives of the Institute of Pathology of the University of Regensburg. Ethics approval was obtained from the ethics committee of the University of Regensburg (reference number: 12-101-0009 and addendum 12-9_1-101).

### Evaluation of histopathological growth patterns

For histologic analysis, one representative hematoxylin and eosin (H&E) stain per metastasis was performed on an automated stainer (HistoCore SPECTRA ST, Leica Biosystems, Nussloch, Germany) according to the standard protocol of the Institute of Pathology of the University of Regensburg. Sections were prepared from all available individual metastases of any patient. Two board certified pathologists (FK and KE) independently assessed the HGP according to the updated consensus guidelines for the scoring of HGP of liver metastases [3]. When discrepancies in HGP scoring occurred, the samples were discussed between the two observers to reach a consensus score. When more than one HGP was present, the percentage proportion of evaluable interface between metastasis and liver parenchyma was assessed for each individual HGP. CRCLM with only one HGP were defined as pure HGP. Non-pure dHGP refers to all HGPs except pure dHGP (“Rotterdam cut-off”). Predominant HGP was defined as the HGP that accounted for more than 50% of the metastasis-liver parenchyma interface in CRCLM with more than one pattern. Mixed pattern was defined as a combination of two HGPs each accounting for 50% of the total interface (i.e. no predominant pattern). If the HGP could not be assessed by H&E staining alone, an additional elastic fiber stain (van Gieson's stain), silver stain (Gomori's stain) and/or immunohistochemical stains (CK7, CK20, HepPar1) were added as needed.

### Immunohistochemical analyses

For immunohistochemistry (IHC), 2 µm thick sections were deparaffinized in xylol, rehydrated through a graded series of ethanol and then stained on an automated IHC stainer (BenchMark ULTRA, Roche, Basel, Switzerland) according to the manufacturer's instructions and standard protocols of the Institute of Pathology, University of Regensburg. The OptiView DAB IHC Detection Kit (Roche, Basel, Switzerland) and the UltraView Universal Alkaline Phosphatase Red Detection Kit (Roche, Basel, Switzerland) were used as detection kits for diaminobenzidine (DAB)/brown staining and red staining, respectively. The following primary antibodies were used: CK7 (mouse monoclonal anti-human antibody clone OV-TL 12/30, Dako Denmark A/S, Glostrup, Denmark, 1:400), CK20 (mouse monoclonal anti-human antibody clone K_5_ 20.8, Dako Denmark A/S, Glostrup, Denmark, 1:250), HepPar1 (mouse monoclonal anti-human antibody clone OCH1E5, Dako Denmark A/S, Glostrup, Denmark, 1:200), MLH1 (purified mouse anti-human antibody clone G168-15, BD Biosciences, Franklin Lakes, New Jersey, USA, 1:20), MSH2 (mouse monoclonal anti-human antibody clone G219-1129, Roche, Basel, Switzerland, ready-to-use), MSH6 (purified mouse anti-human antibody clone 44, BD Biosciences, Franklin Lakes, New Jersey, USA, 1:300), and PMS2 (mouse monoclonal anti-human antibody clone A16-4, Roche, Basel, Switzerland, ready-to-use). For the evaluation of mismatch-repair-status, expression of all four mismatch-repair-proteins (MLH1, MSH2, MSH6, and PMS2) was assessed by IHC. Retained expression was defined as positive staining in at least 10% of tumor cell nuclei. In tumors with unevaluable IHC in any of the four mismatch-repair-proteins, microsatellite-analysis by PCR was additionally performed.

### DNA isolation for *KRAS-*, *NRAS-* and *BRAF*-mutation profiling and microsatellite PCR

To isolate DNA from the FFPE tissue, the samples were sectioned into 5 µm-thick slices and a microdissection guided by the H&E (tumor region marked by a board-certified pathologist) and methylene blue counterstaining was performed. DNA was extracted using the Maxwell RSC Blood DNA Kit and the Maxwell CSC 48 (both Promega Corporation, Madison, Wisconsin, USA). DNA concentration was measured with a Qubit 4 fluorometer (Thermo Fisher Scientific, Waltham, Massachusetts, USA).

### Microsatellite PCR

Tumor DNA and corresponding normal DNA were subjected to microsatellite PCR using the so-called Bethesda panel comprising two mononucleotide (BAT25 and BAT26) and three dinucleotide markers (D5S346, D2S123, and D17S250) [17]. Briefly, 20–50 ng DNA was used to amplify the microsatellite loci in multiplex PCR reactions. PCR products were separated by capillary electrophoresis using a SeqStudio Genetic Analyzer (Applied Biosystems, Waltham, Massachusetts, USA). A tumor was classified as having high-level microsatellite instability (MSI-H) when two or more markers of the Bethesda panel showed an allelic size variation (i.e., a band shift compared with corresponding normal DNA), as having low-level microsatellite instability (MSI-L) when one marker showed an allelic size variation, and as microsatellite stable (MSS) when no marker showed an allelic size variation.

### *KRAS*-, *NRAS*- and *BRAF*-mutation testing by melting curve analysis and pyrosequencing

The molecular characterization of *RAS* (*KRAS*/*NRAS*) and *BRAF* genetic hotspots (exons 2 to 4 of *KRAS*/*NRAS* and exon 15 of *BRAF*, respectively) was conducted using quantitative real-time PCR (qPCR) in combination with High Resolution Melting (HRM) analysis on a LightCycler480 (Roche, Basel, Switzerland). About 20 ng of template DNA was used. In case HRM showed a melting curve that was unclear or indicative of a mutation, pyrosequencing was additionally performed using the PyroMark Q24 system (Qiagen N.V., Venlo, The Netherlands).

### Microdissection and RNA extraction

**Fig. 2:**
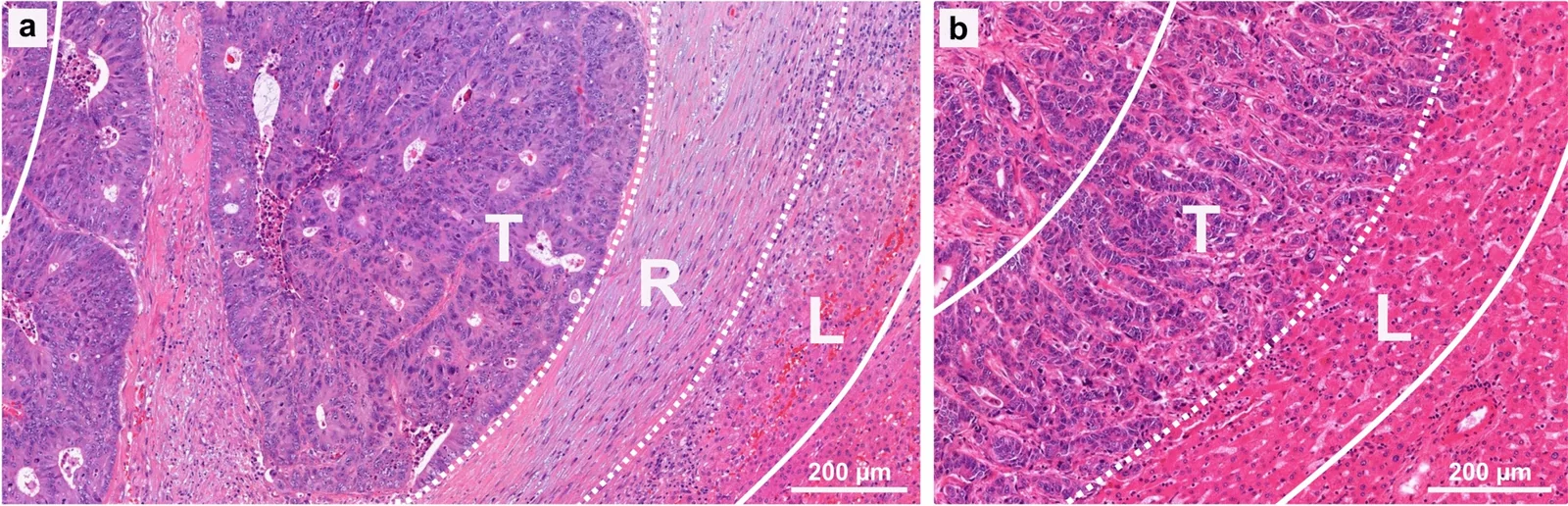
Histopathology (H&E) illustrates the areas selected for microdissection. **a** In dHGP, the microdissected region (area between the two solid white lines) consisted of tumor tissue (T), accounting for approximately two-thirds of the sampled area, and the fibrotic rim (R) together with adjacent liver parenchyma (L), accounting for the remaining one-third. **b** In rHGP and pushing HGP, where no fibrotic rim is present, the microdissected area comprised approximately two-thirds tumor tissue and one-third adjacent liver parenchyma. The boundaries between the different tissue types within the microdissected regions are indicated by dotted white lines

FFPE samples were used for RNA isolation (3–6 sections of 10 µm thickness, depending on the sample size). To encompass the entire MMPI area, microdissection included tumor tissue, the fibrotic rim (in dHGP), and adjacent liver parenchyma. In dHGP, the microdissected area was composed of tumor tissue (approximately two-thirds of the microdissected area) and the fibrotic rim including adjacent liver parenchyma (approximately one-third of the microdissected area). In rHGP and pushing HGP, where a fibrotic rim is absent, tumor tissue and adjacent liver parenchyma accounted for approximately two-thirds and one-third of the microdissected area, respectively. In CRCLM exhibiting non-pure HGPs, microdissected regions were selected to reflect the relative proportions of the different HGPs present. Figure 2 illustrates the microdissected areas for dHGP (Fig. 2a) and rHGP (Fig. 2b). RNA extraction was performed manually using the RNeasy FFPE Kit (Qiagen N.V., Venlo, The Netherlands) according to the manufacturer's instructions. RNA quantity and integrity were assessed by electrophoresis on a 4200 TapeStation system (Agilent, Santa Clara, USA), and RNA purity ratios (260/280 and 260/230) were measured for each sample using a DS-11 spectrophotometer/fluorometer (DeNovix Inc., Wilmington, USA).

### Gene expression analysis

Gene expression analysis was performed using the NanoString nCounter® XT assay (NanoString Technologies, Seattle, USA) according to the manufacturer's instructions. Briefly, a minimum of 100 ng of RNA (range 100–300 ng) was hybridized with the NanoString nCounter® PanCancer Progression Panel code set, which includes 740 target genes and 30 reference genes (supplementary table S1). Following the manufacturer’s instructions, samples were automatically purified and immobilized on the sample cartridge using the NanoString PrepStation and transferred to the nCounter® Digital Analyzer for data acquisition.

We assayed the expression of 770 genes using the NanoString nCounter® PanCancer Progression Panel. RNA expression data were normalized using the housekeeping gene-based RUVseq normalization approach, described in detail by Bhattacharya et al. [18] with the R package RUVseq. Briefly, samples were first checked for compliance with technical quality control criteria, analogous to the quality control check of the nSolver software (NanoString Technologies, Seattle, USA) and further evaluated based on below-limit-of-detection measurements. Housekeeping genes most suitable for normalization were then identified from the 30 reference genes included in the PanCancer Progression Panel (supplementary table S1). We then performed RUVseq normalization and confirmed satisfactory results by means of relative log expression (RLE)-plots. Differential gene expression analysis (DGEA) was performed using the DESeq2 R package [19] including RUV factors in the design formula as suggested by [18].

Principal Component Analysis (PCA) was performed on log-normalized counts of the 500 most variable genes. Shaded areas were drawn around the central 75% of data points for each group by ellipses representing bivariate t-distributions on the PC1–PC2 plane, summarizing within-group dispersion and overlap.

### Gene set enrichment analysis

2440 gene sets from 6 collections of the Molecular Signature Database (MSigDB) (hallmark gene sets, C2 WikiPathways [20], C4 Curated Cancer Cell Atlas [21], C4 cancer modules [22], C6 oncogenic gene sets, C8 cell type signature, accessed June 2024) [23, 24] were screened for overlap with the NanoString nCounter® PanCancer Progression Panel code set. Only gene sets with a relative overlap with the NanoString nCounter® PanCancer Progression Panel of at least 33.3% and an absolute overlap of at least 10 genes were preselected. We then manually excluded 31 gene sets to ensure a maximum overlap of 60% between the gene sets, prioritizing more recent publications and greater NanoString panel overlap. To the resulting list of 120 gene sets, we added 10 further gene sets with limited but acceptable overlap to enable comparisons with previously published literature (in brackets: previous literature followed by absolute overlap of the NanoString nCounter® PanCancer Progression Panel and the gene set): From the hallmark gene sets, we added complement, glycolysis, IL6/JAK/STAT3 signaling, inflammatory response, interferon gamma response, KRAS signaling up, and TNF-alpha signaling via NFKB, as these gene sets were also examined by Latacz et al. [13] and Hu et al. [12] (absolute overlap with the NanoString nCounter® PanCancer Progression Panel: complement 39/200, glycolysis 29/200, IL6/JAK/STAT3 signaling 23/87, inflammatory response 42/200, interferon gamma response 25/200, KRAS signaling up 35/200, and TNF-alpha signaling via NFKB 36/200). From the Kyoto Encyclopedia of Genes and Genomes (KEGG) [25], we added the WNT signaling pathway and glycolysis/gluconeogenesis, as these gene sets were also examined by Fleischer et al. [11] (absolute overlap: WNT signaling pathway 36/151, glycolysis/gluconeogenesis 11/62). We further added the core angiogenesis gene set defined by Masiero et al. [26], as analyzed in Latacz et al. [13] (absolute overlap: 8/20). Gene Set Enrichment Analysis (GSEA) was carried out using the Fast Gene Set Enrichment Analysis (fgsea) package on R version 4.4 [27] comparing the final selection of 130 gene sets with a ranked list of genes from the differential gene expression analysis of pure dHGP metastases vs. non-pure dHGP metastases. Ranking was performed by placing genes that were consistently upregulated in pure dHGP metastases at the top of the list and genes that were consistently upregulated in all other metastases at the bottom of the list using the Wald statistic (log_2_(foldchange) value divided by its standard error).

### Statistical analysis

Descriptive analyses included absolute (n) and percentage (%) frequencies for categorical variables and mean (m) and standard deviation (SD) or median (med) and quartiles as well as minimum and maximum for continuous variables.

For the entire cohort, the reverse Kaplan–Meier method was used to calculate median follow-up time (including 95% confidence interval (CI)). Overall survival was estimated from metastasectomy of the first evaluable liver metastasis to death or censoring. First, log-rank tests were used to compare survival between HGPs. The following HGP definitions were evaluated: (1) "Rotterdam cut-off" [3]: pure dHGP vs. all other HGPs (stratified by extent of non-desmoplastic HGP or grouped into other HGP categories) and (2) predominant HGP. SPSS software (version 29.0.1) was used for this statistical analysis.

In the subcohort analyzed for gene expression by NanoString, we assessed per-patient overall survival after first metastasectomy by fitting multivariate Cox proportional hazards models (coxph function from R's survival package, default parameters). The main covariate of interest was patient-level HGP, grouped either as pure dHGP versus non-pure dHGPs (n=49) (see section “Evaluation of histopathological growth patterns”) or as pure or predominant dHGP vs. pure or predominant rHGP (n = 47). Models were additionally adjusted for age at diagnosis (continuous), sex (categorical), tumor grade (2 vs. 3, categorical), and *RAS*/*BRAF* mutation status (wild-type vs. mutated, categorical).

The survival analysis with gene expression included 52 patients for which both overall survival and gene expression data were available. For patients with multiple sampled metastases, we prioritized infiltrative metastases (pure rHGP > predominant rHGP > predominant dHGP > pure dHGP) with later sampling timepoints. In 3 patients where ties remained, we averaged the gene expression profiles. As gene expression features, we tested log-transformed and normalized counts of the 55 genes significantly differentially expressed (adjusted *P*-value (*p*_adj_) < 0.05 and minimum fold change of 150% in either direction) between pure dHGP CRCLM and all others. Additionally, we tested all 750 possible gene-pair ratios with 25 pure dHGP-overexpressed genes in the numerator and 30 non-pure dHGP-overexpressed genes in the denominator. For each feature, we fitted a bivariate Cox proportional hazards model (coxph function of R's survival package, with default parameters), controlling for HGP group.

## Results

### Cohort characteristics

**Fig. 3:**
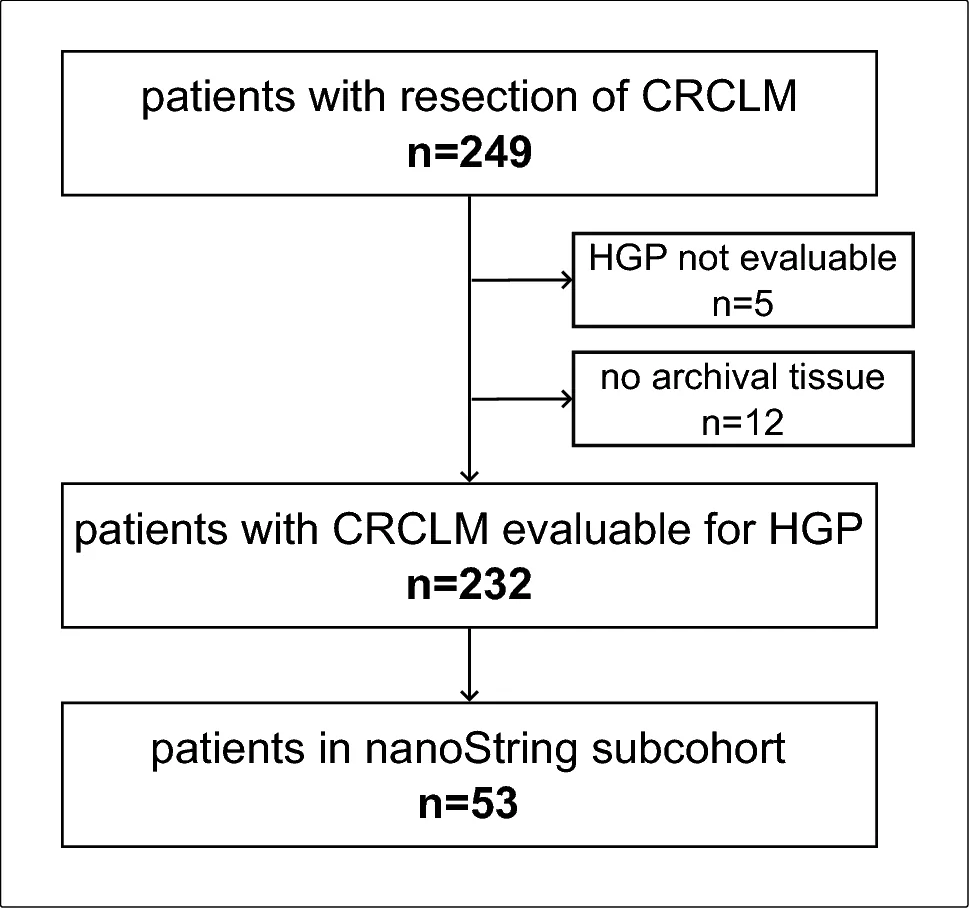
The flow diagram depicts the selection of patients from the large cohort to the final subcohort subjected to gene expression analysis by NanoString

Of the 249 patients selected from a retrospective cohort who underwent surgical resection of CRCLM at University Hospital Regensburg between February 2003 and January 2016, 232 had CRCLM specimens with available FFPE tissue and which were evaluable for HGP. In total, 495 individual liver metastases were histologically evaluated for HGP. A subcohort of 53 patients was subsequently selected for NanoString analysis (Fig. 3). A summary of the characteristics of the entire cohort is provided in supplementary table S2. According to the updated consensus guidelines by Latacz et al. [3], the most prevalent HGPs among individual CRCLM were: pure rHGP (192/495, 38.8%), pure dHGP (104/495, 21.0%), predominant rHGP (96/495, 19.4%), and predominant dHGP (76/495, 15.4%). Considering that 100 patients had more than one CRCLM, the HGP was also determined at the patient-level. For patients with more than one metastasis, the average of the HGP scores of all separate metastases was determined. On the patient level, the most prevalent HGPs were pure rHGP (75/232, 32.3%), predominant rHGP (59/232, 25.4%), predominant dHGP (49/232, 21.1%), and pure dHGP (36/232, 15.5%). The HGP distributions of the entire cohort and the histological features of the most prevalent HGPs (dHGP, rHGP) are depicted in supplementary figure S1.

Follow-up information was available for 153 patients (153/232, 65.9%) with a median follow-up time of 147.0 months (95% CI 136.0–158.0, range 1–172). 43 of 153 patients (28.1%) were alive at last follow-up and 110 of 153 patients (71.9%) had died, with a median overall survival after first metastasectomy of 50.0 months (95% CI 37.0–60.0). Using the “Rotterdam cut-off”, overall survival was significantly longer for patients with pure dHGP (n = 23) vs. non-pure dHGP (n = 130) (*p* < 0.001). Kaplan–Meier survival curves comparing different HGP categorizations are shown in supplementary figure S2.

### Subcohort for gene expression analysis

For gene expression analysis, a subcohort was selected of patients that approximately reflected the HGP distribution of the entire cohort, while also requiring availability of sufficient FFPE tissue, adequate tumor-liver interface for reliable microdissection, and inclusion of patients with available primary tumors and/or multiple metastases to enable exploratory analysis of intra-patient variability. The subcohort encompassed 128 samples (106 CRCLM resected from February 2003 to November 2012, and 22 corresponding primary colon cancer samples) of 53 patients. In 26 patients, one CRCLM was analyzed, whereas in the remaining patients, multiple CRCLM from synchronous and/or metachronous metastasectomies were included to ensure that patient-intrinsic factors were not excluded from further analyses.

Five patients had known activating *KRAS*-mutations from prior diagnostic work-up. For all remaining cases, *KRAS*/*NRAS*/*BRAF*-mutation testing was additionally performed. In total, 24 patients (45.3%) had *RAS*/*BRAF*-mutated tumors: 19 patients (35.8%) had activating *KRAS* mutations, four patients (7.5%) had activating *NRAS* mutations, and one patient (1.9%) had a *BRAF* V600E mutation and a truncating *KRAS* Q61Ter mutation, which was reproducible after repeated DNA extraction and mutation analysis. This patient had a locally advanced mucinous adenocarcinoma of the right colonic flexure. There were no morphologically distinct tumor areas in the liver metastasis. One patient (1.9%) was not evaluable for mutation status because of poor DNA quality. All tumors were mismatch-repair-proficient or microsatellite-stable. We tested whether *KRAS*/*NRAS*/*BRAF* mutation status (dichotomized into wild-type vs. mutated) was associated with HGP using Fisher's exact tests at the patient level. For pure dHGP versus non-pure dHGP (n = 52), we observed a non-significant trend toward enrichment of wild-type tumors in pure dHGP (odds ratio 2.23; *p* = 0.309). A similar, weaker trend was observed when comparing pure or predominant dHGP versus pure or predominant rHGP (n = 50; odds ratio 1.60; *p* = 0.572).

The distribution of HGPs and additional clinicopathological characteristics are summarized in Table 1. A detailed timeline illustrating primary tumor resection, systemic therapies, and the resection of CRCLM specimens analyzed by NanoString is provided in supplementary figure S3.

**Table 1:** Clinicopathological characteristics of the NanoString subcohort

Number of patients	n = 53
*Sex*	
Female	17 (32.1%)
Male	36 (67.9%)
Median age at primary diagnosis (years)	57 (IQR: 50.5–65)
*Primary tumor site*	
Cecum and ascending colon	7 (13.2%)
Right colonic flexure to sigmoid colon	18 (34.0%)
Rectum	19 (35.8%)
N/A	9 (17.0%)
*UICC stage at primary diagnosis*	
Stage I	1 (1.9%)
Stage II	7 (13.2%)
Stage III	8 (15.1%)
Stage IV	30 (56.6%)
N/A	7 (13.2%)
*Primary tumor grade*	
G1	0
G2	39 (73.6%)
G3	11 (20.8%)
N/A	3 (5.7%)
*Mutation/mismatch-repair status*	
*KRAS* mutation	19 (35.8%)
*NRAS* mutation	4 (7.5%)
*BRAF* and *KRAS* mutation	1 (1.9%)
Mutation status not evaluable	1 (1.9%)
*KRAS/NRAS/BRAF* wild-type	28 (52.8%)
MMRp/MSS	53 (100%)
*CRCLM analyzes by NanoString*	n = 106
CRCLM per patient (median)	2 (IQR: 1–2)
Patients with synchronous CRCLM analyzed	19 (35.8%)
Patients with metachronous CRCLM analyzed	17 (32.1%)
Patients with synchronous and metachronous CRCLM analyzed	9 (17.0%)
*HGP of CRCLM analyzed by NanoString*	
Pure dHGP	27 (25.5%)
Predominant dHGP	22 (20.8%)
Predominant rHGP	18 (17.0%)
Pure rHGP	32 (30.2%)
Predominant pushing HGP	7 (6.6%)
*CRCLM after systemic therapy*	
Any systemic therapy within 365 days prior to CRCLM resection	27 (25.5%)
Anti-VEGF therapy within 365 days prior to CRCLM resection	3 (2.8%)

### Multivariate survival analysis of NanoString subcohort

**Fig. 4:**
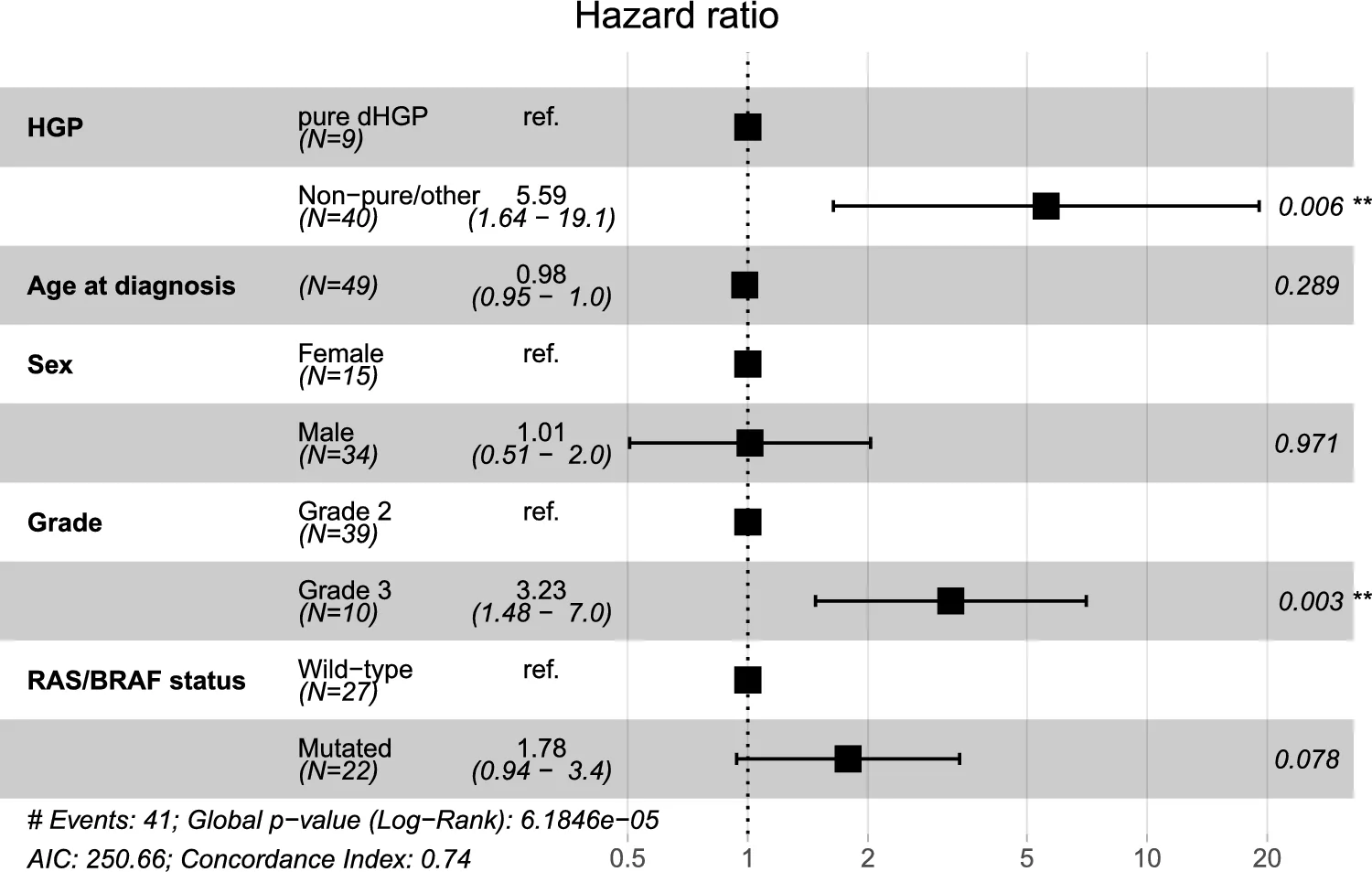
The Cox proportional hazards model shows the association between patient-level HGP and overall survival after first metastasectomy. After dichotomizing HGP into pure dHGP and non-pure dHGP, non-pure dHGP was significantly associated with shorter overall survival. Higher tumor grade was also significantly associated with shorter overall survival, while *KRAS*/*NRAS*/*BRAF* mutation status showed only a non-significant association with poorer outcome. **Indicates significant results at *p* < 0.05

52 of 53 patients in the NanoString subcohort had available follow-up data with a median follow-up time of 163.7 months (95% CI 143.2–not reached; range 1.4–187.3). 10 of 52 were alive at last follow-up and 42 patients had died. The median overall survival after first metastasectomy was 43.2 months (95% CI 28.9–64.3) for the NanoString subcohort, similar to the 50.0 months (95% CI 37.0–60.0) of the overall cohort. To investigate the association between patient-level HGP and overall survival after first metastasectomy in the NanoString subcohort, we fitted a Cox proportional hazard model (Fig. 4). As the main covariate of interest, we included patient-level HGP, dichotomized as per "Rotterdam cut-off" (pure dHGP vs. non-pure dHGPs). We adjusted for patient's age at diagnosis, primary tumor grade and *RAS*/*BRAF* mutation status (n = 49 with complete data available). In line with previous literature [3, 10], we found that any HGP other than pure dHGP was strongly associated with adverse outcome (hazard ratio 5.59; 95% CI 1.64–19.1; *p* = 0.006). Additionally, a higher grade was significantly associated with adverse outcome (hazard ratio 3.23; 95% CI 1.48–7.0; *p* = 0.003). We repeated this analysis, only changing the HGP dichotomization to pure or predominant dHGP vs. pure or predominant rHGP (n = 47). The resulting Cox model was qualitatively very similar in terms of the adjustment covariates, but showed a weaker and non-significant association for HGP group (HR for pure or predominant rHGP: 1.95, 95% CI 0.97–3.92, *p* = 0.062).

### Principal component analysis of gene expression

**Fig. 5:**
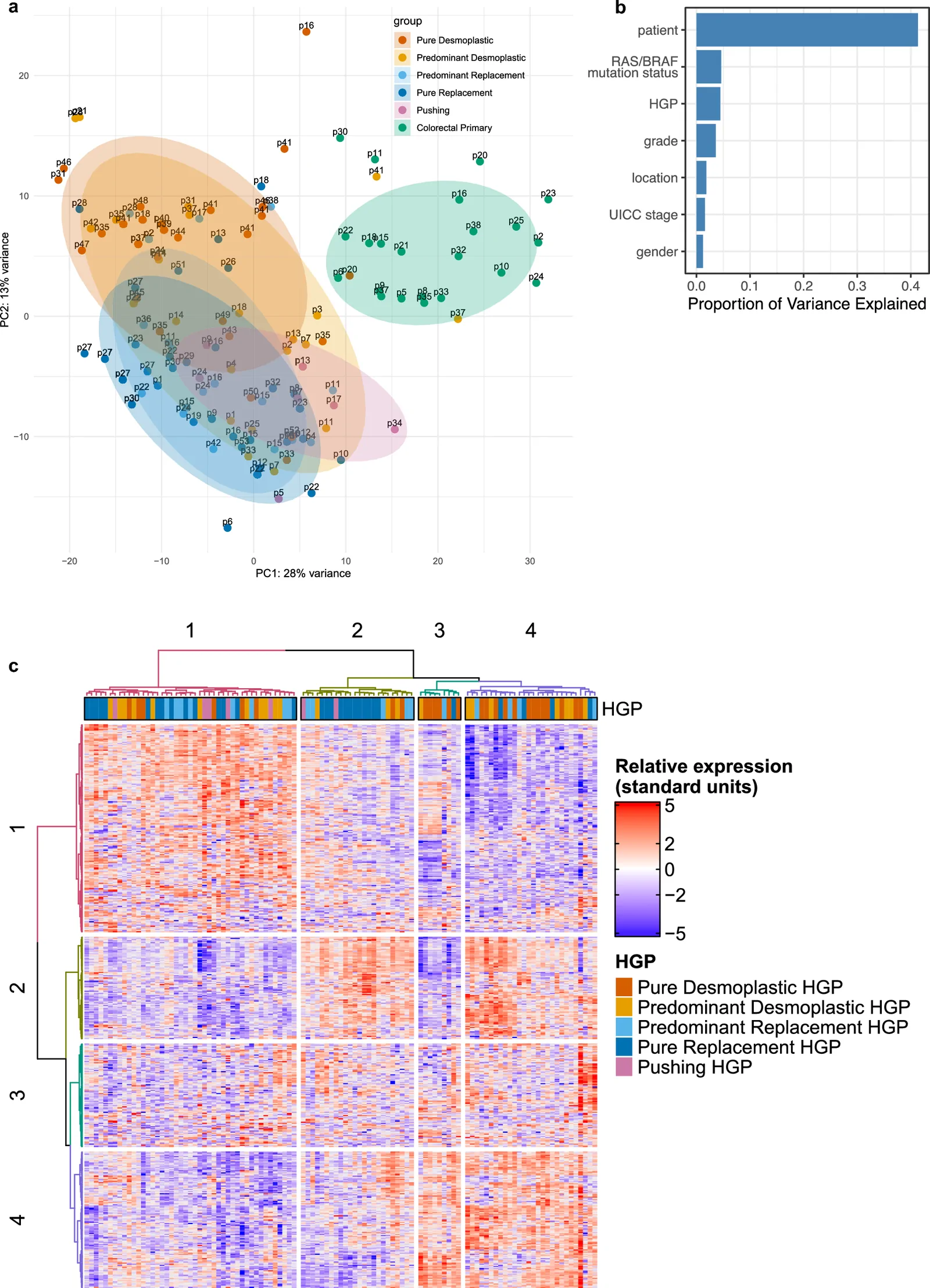
Global gene expression variance is driven by tumor site (liver vs. colorectal primary) and patient-specific factors rather than HGP. **a** PCA plot accounting for 41% of the variance in gene expression of CRCLM and colorectal primary tumors with segregation of primary tumors from CRCLM (ellipses corresponding to estimated multivariate t-distributions that cover approximately 75% of observations of each group, stat_ellipse (type = "t") in ggplot2). **b** PVCA of CRCLM pointing out patient ID (i.e. patient- and tumor-specific factors) as most important covariate followed by *KRAS*/*NRAS/BRAF*-mutation status, HGP, and primary tumor grade. Only minimal impact of gender, UICC stage at diagnosis and primary tumor location (i.e. right-sided colon, left-sided colon, and rectum) on gene expression variance. **c** Heatmap showing unsupervised clustering of the 500 most variably expressed genes by CRCLM with clusters mainly driven by other factors than HGP

To obtain a global overview of gene expression across our cohort, we performed a principal component analysis (PCA) on the normalized expression of all genes across all samples (Fig. 5a). The first principal component (PC1) accounted for 28.0% of the total variance and roughly separated the 22 primary colon cancer samples from the CRCLM samples. The second principal component (PC2) explained 13.0% of the total variance and showed a loose trend in which dHGP samples tended to separate from rHGP and pushing HGP samples along this axis.

### Contribution of patient- and tumor-specific factors to gene expression variance

To quantify the relative contribution of the HGP group and other covariates to the overall gene expression variation, we performed principal variance component analysis (PVCA) on the normalized, log-transformed counts of all CRCLM samples (Fig. 5b). PVCA revealed that the HGP group was an important source of variance in CRCLM gene expression but was outranked by the variable patient identity (ID). Tumor grade as well as *RAS*/*BRAF* mutation status also contributed substantially to variance in the overall gene expression, whereas patient gender, Union for International Cancer Control (UICC) stage and primary tumor location contributed minimally (Fig. 5b).

### Unsupervised clustering of the most variably expressed genes

We then performed unsupervised clustering on the 106 CRCLM samples using standardized expression values of the 500 most variable genes (Fig. 5c). Clustering was based on the Manhattan distance metric and the Ward’s clustering method using the *dist* and *hclust* functions within the R Stats package. Both genes and samples were partitioned into k = 4 clusters as determined by silhouette score analysis across possible k values ranging from 2 to 10. Cluster 1 (red dendrogram) exhibited relatively homogeneous gene expression profiles but consisted of diverse HGP types. Only pure dHGP was significantly depleted (*p* = 0.015), whereas no other HGP type was significantly over- or underrepresented. In contrast, samples outside cluster 1 were more heterogeneous in their overall gene expression and were further subdivided into clusters 2, 3 and 4. Clusters 2 and 4 were of comparable size but different in their HGP composition, with a significant enrichment for pure dHGP in cluster 4 (*p* = 0.005), and a significant enrichment for pure rHGP in cluster 2 (*p* = 0.023), with reciprocal significant depletions vice versa. The distinctiveness of the smallest cluster 3 may be explained by patient- and tumor-specific patterns, with 5 of its 9 samples originating from the same patient (patient ID: p41). To gain insight into the biological features of the identified gene expression clusters, we performed overrepresentation analysis using the *fgsea* package on a curated panel of 120 gene sets selected for compatibility with our targeted assay (see Methods). Cluster 4 showed enrichment for a broad range of gene sets associated with stromal components including strong overrepresentation of the Metaprogram 1 of Cancer-associated fibroblasts [21] (p_adj_= 1.0*10^–7^). Consistent with this, cluster 4 contained many genes encoding collagens (*COL1A1*, *COL1A2*, *COL3A1*, *COL5A1*, *COL6A1*, *COL7A1*). In cluster 1, we observed significant overrepresentation of the α6β4 integrin signaling pathway (*p*_adj_=0.0024), as evidenced by the presence of multiple laminin genes (*LAMA1*, *LAMA3*, *LAMA5*, *LAMB3*, *LAMC2*).

The overrepresentation analysis did not yield significant enrichment for any of the 120 gene sets in gene cluster 2 however, several genes in this cluster are typically expressed by liver parenchyma (*ALB*, *APOE*, *APOH*, *TF*, *AGT*, *HGF*), suggesting an important liver tissue signal in cluster 2.

### Differential gene expression analysis between pure dHGP and all other HGPs

**Fig. 6:**
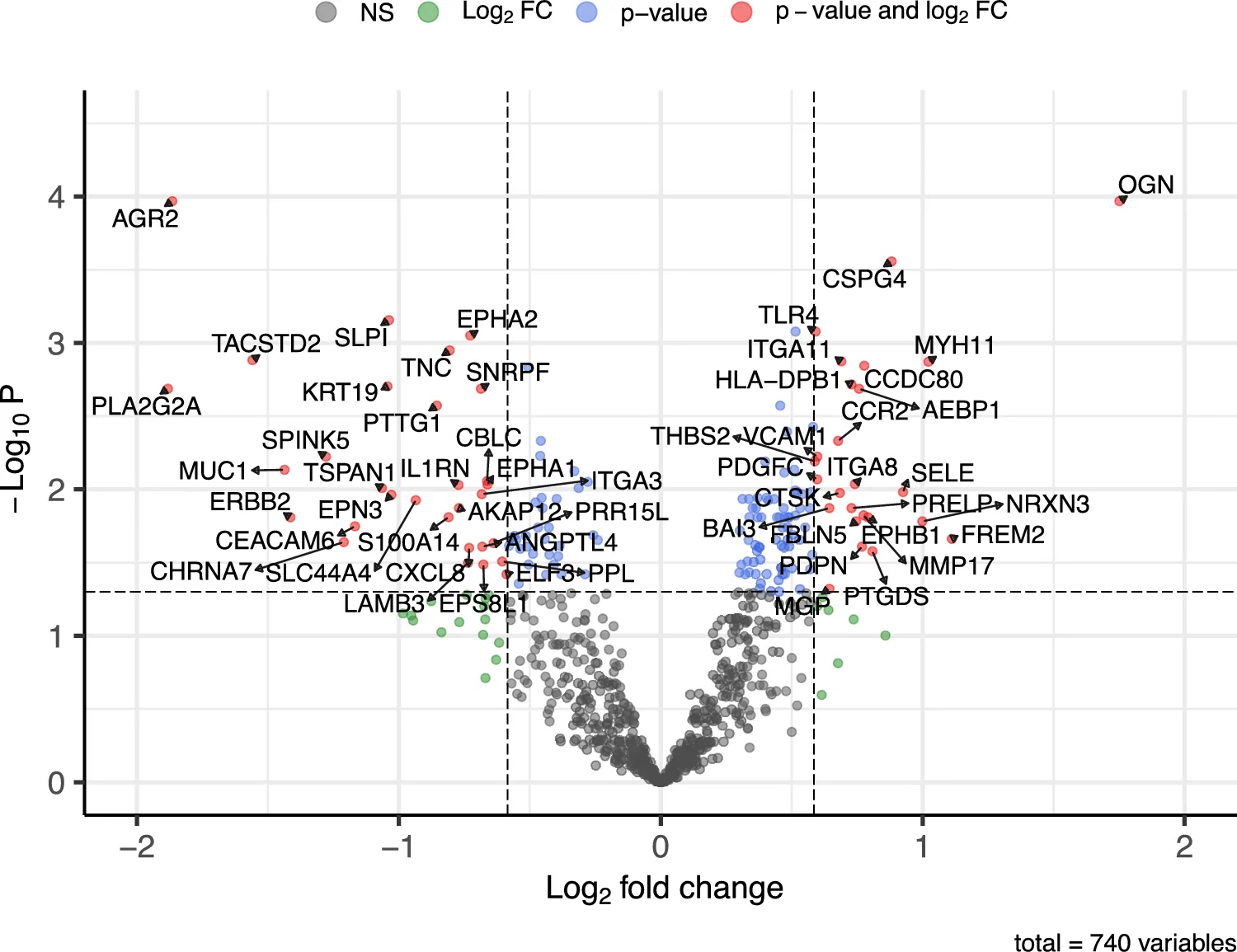
CRCLM with pure dHGP show a distinct transcriptional profile compared to other HGP types. Volcano plot of differentially expressed genes between CRCLM with pure dHGP and all other types of HGP shows significantly upregulated genes (*p*_adj_ < 0.05 and log_2_(fold change) > 0.585) in pure dHGP in the upper right corner and significantly upregulated genes in all other types of HGP in the upper left corner. FC = fold change

Given the significantly better prognosis associated with pure dHGP of CRCLM compared to other HGP subtypes, we sought to characterize differences in gene expression between pure dHGP and all other HGP subtypes. Evidence from the PCA and unsupervised clustering analyses, particularly the significant enrichment of pure dHGP samples in cluster 4, already suggested distinct transcriptomic profiles between these groups. To specifically investigate these differences, we conducted differential gene expression analysis using DESeq2, comparing pure dHGP CRCLM samples against all other HGP subtypes. Since patient- and tumor-specific signals proved to be strong contributors to variance in gene expression in CRCLM, we used two test strategies to account for patient- and tumor-specific effects in this comparison:

Strategy 1: For patients with multiple samples belonging to the same HGP group, we first averaged the normalized expression values across those samples to obtain a single representative profile per HGP group per patient prior to testing. This reduced intra-patient redundancy and minimized overrepresentation of individual patients. Strategy 2: We restricted data to those 9 patients that had at least one sample on each side of the comparison (pure dHGP versus all other HGP subtypes, 30 total samples) and then modeled differential gene expression with patient ID as a covariate. At *p*_adj_ < 0.05 and a minimum fold change of 150% in either direction, strategy 1 identified 25 genes significantly upregulated and 30 genes significantly downregulated in pure dHGP compared to all others (Fig. 6). *AGR2* was re-identified as significantly downregulated in pure dHGP using strategy 2 with the same thresholds (supplementary fig. S4). The results of both strategies were strongly correlated when sorted by Wald statistic, indicating that the core trends were consistent (Spearman’s ρ = 0.69, *p* < 2.2 × 10⁻^16^). However, the fold change and *P*-value distributions of strategy 2 suggest that it was likely underpowered (supplementary fig.S4). Taking this into account, we aimed to propose a set of genes with differential expression trends consistent across both strategies, i.e. genes which may be robustly associated with HGP across diverse patient-specific gene expression backgrounds. To facilitate this, we identified genes that exceeded our effect size thresholds (fold change of 150% in either direction) with a consistent effect direction in both analyses, but without applying the *P*-value threshold of *p*_adj_ < 0.05. This approach yielded sets of 11 genes on each side of the comparison. We then sorted both sets by the rank product of the Wald statistic from both analyses (provided as supplementary tables S3 and S4). The top hits produced were *ITGA8*, *VCAM1* and *CCR2* for pure dHGP and *AGR2*, *SNRPF* and *PTTG1* for non-pure dHGP.

### Gene set enrichment analysis

To identify functional patterns among the differentially expressed genes between pure dHGP and all other HGP subtypes, we performed Gene Set Enrichment Analysis (GSEA) using the 130 preselected gene sets described earlier on the Wald statistic rankings derived from strategy 1.

### Gene sets enriched in pure dHGP

**Fig. 7:**
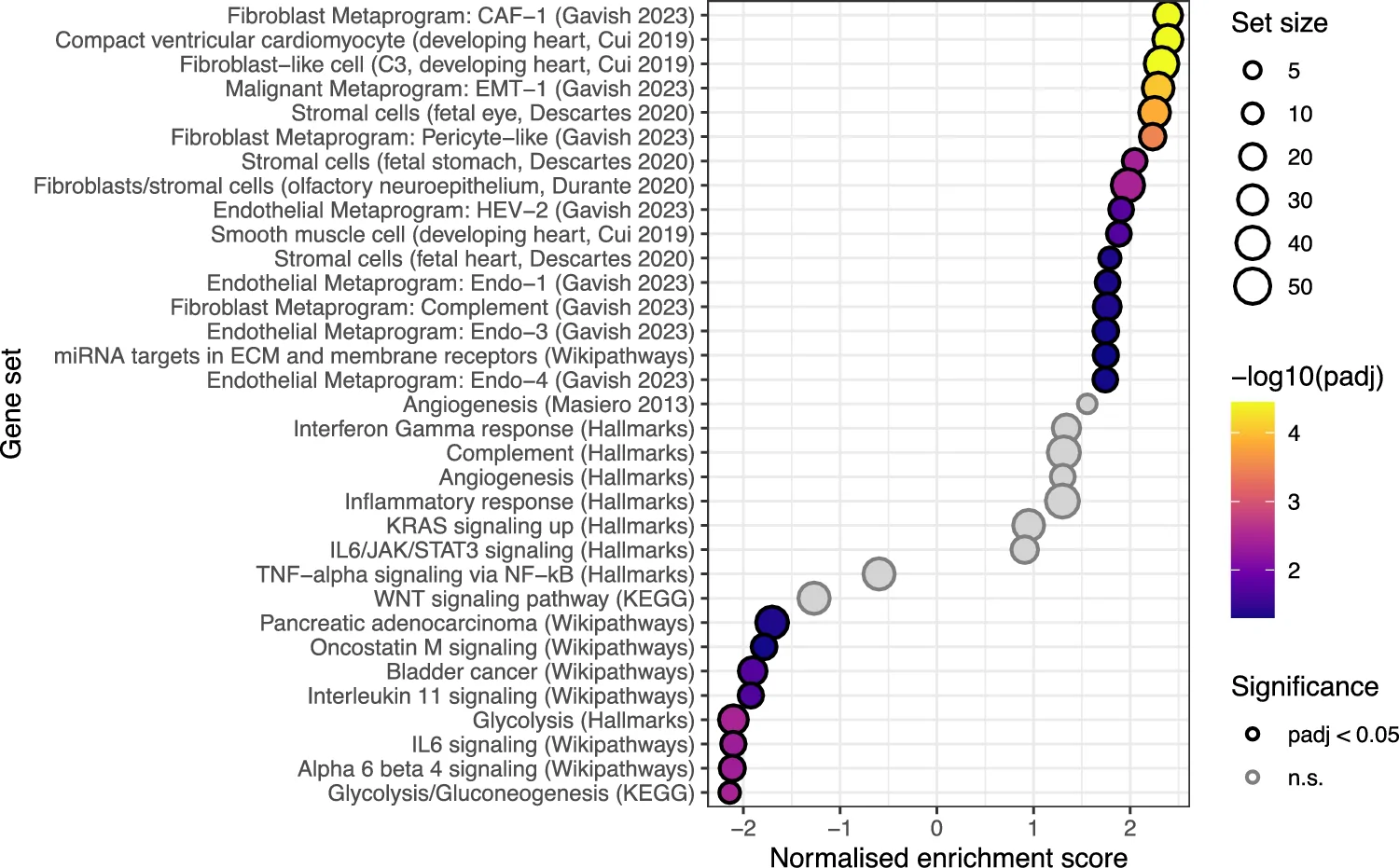
Gene set enrichment analysis reveals enrichment of extracellular matrix organization gene sets in pure dHGP and α6β4 integrin signaling, glucose metabolism and interleukin signaling gene sets in non-pure dHGP. Gene sets significantly enriched (*p*_adj_ < 0.05) in genes differentially expressed between CRCLM with pure dHGP and all other types of HGP are shown. Positive normalized enrichment scores (NES) indicate enrichment in genes overexpressed in pure dHGP metastases. Set size refers to the absolute overlap between the gene set and the NanoString nCounter® PanCancer Progression Panel

**Fig. 8:**
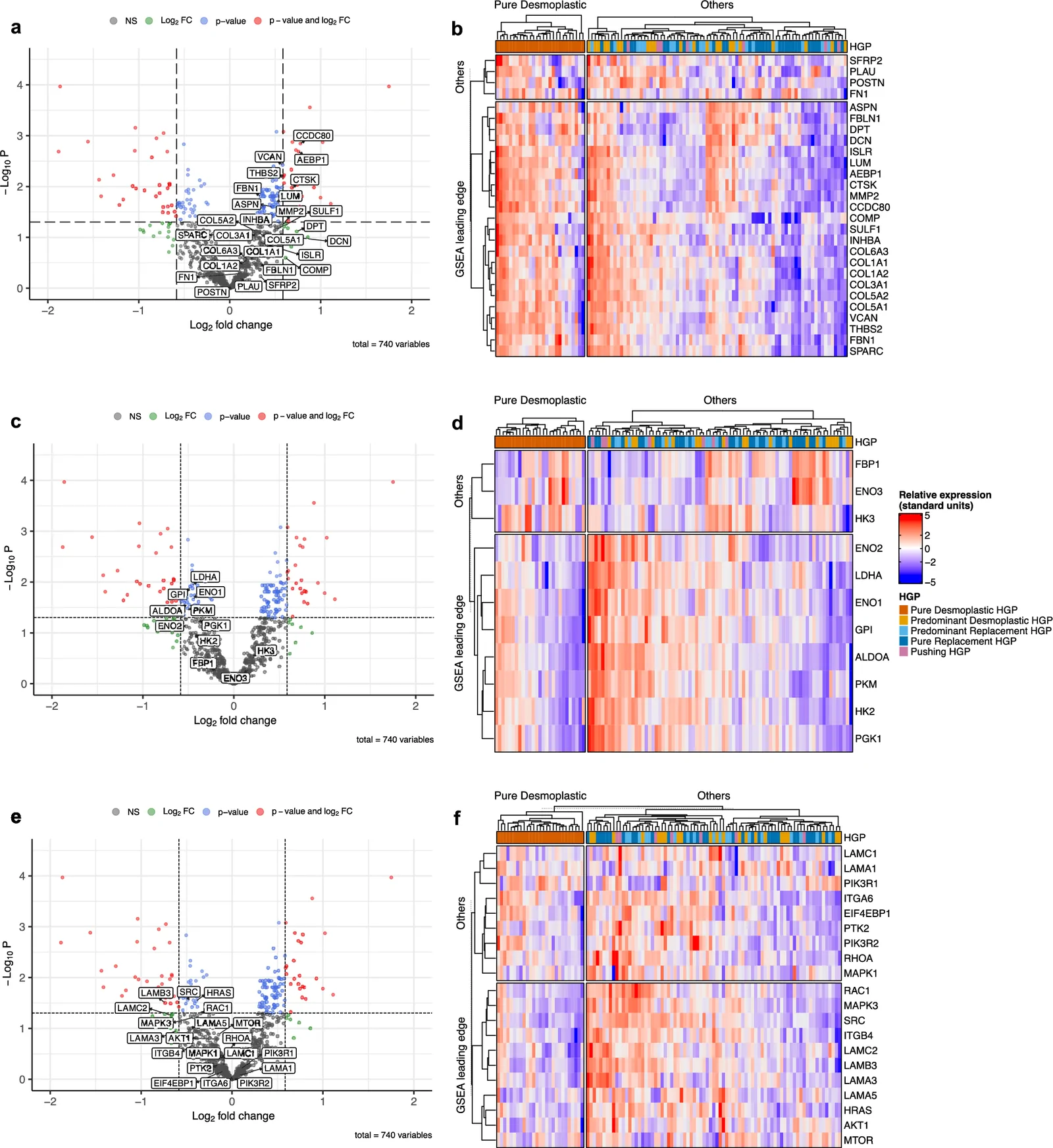
Gene sets associated with ECM organization are enriched in pure dHGP (exemplified by Metaprogram 1 of Cancer-associated fibroblasts [21]), whereas the gene sets glycolysis/gluconeogenesis (KEGG) and α6β4 integrin signaling (Wikipathways) are enriched in non-pure dHGP. Volcano plots highlighting genes in these gene sets that overlapped with the NanoString panel and were differentially expressed between pure dHGP and non-pure dHGP CRCLM, with corresponding heatmaps of all overlapping genes in the respective gene sets (leading edge separated from all other genes in the gene set): **a**,** b** gene set Metaprogram 1 of cancer-associated fibroblasts, **c**, **d** glycolysis/gluconeogenesis gene set, **e**, **f** α6β4 integrin signaling

GSEA revealed an enrichment of multiple gene sets involved in extracellular matrix (ECM) organization in pure dHGP CRCLM (Fig. 7). For example, enrichment of Metaprogram 1 of Cancer-associated fibroblasts [21] was driven by many collagen-encoding genes (*COL5A2*, *COL5A1*, *COL3A1*, *COL1A1*, *COL6A3*, *COL1A2*) as well as other genes involved in ECM remodeling like *CCDC80*, *AEBP1*, *THBS2*, *CTSK* and *MMP2* (Fig. 8a, b). Further gene sets significantly enriched in pure dHGPs are associated with stromal cells more generally (Fig. 7). These results are consistent with previous reports of signals from stromal cells and specifically fibroblasts in gene expression data of dHGP relative to rHGP [12, 13]. We also report significant enrichment of gene sets reflecting endothelial cells and non-significant enrichment of gene sets relating to angiogenesis, roughly in line with previous literature emphasizing the role of angiogenesis in dHGP [11–13]. Further, our results corroborate previously reported enrichments of EMT gene sets in dHGPs [11, 12]. These studies also report enrichment of gene sets related to inflammatory responses in dHGP [12, 13]; testing the same gene sets, we observed a similar trend, though not statistically significant.

### Gene sets enriched in non-pure dHGP

In contrast, we found gene sets significantly downregulated in pure dHGP compared to other HGPs; these are associated with glucose metabolism, α6β4 integrin signaling and interleukin signaling (Fig. 7). Increased glucose metabolism has been described previously in rHGP compared to dHGP and linked to the heightened energy demands of aggressive proliferation [11–13]. Indeed, we found some key players in glycolysis (e.g., *ALDOA*, *PKM*, *LDHA*) significantly upregulated (p_adj_ < 0.05) in CRCLM with a compromised or absent desmoplastic rim (i.e., non-pure dHGP) as shown for the gene set glycolysis/gluconeogenesis (KEGG) (Fig. 8c, d). Interestingly, the enrichment of interleukin signaling gene sets (i.e., IL6 and IL11 signaling pathways) in this group of metastases is inconsistent with previous reports specifically proposing IL6-STAT3 as an important feature of dHGP [13]. Reviewing the main genes driving this enrichment in our data, however, we found that many are more broadly associated with survival/proliferative signaling rather than an inflammatory cytokine response specifically; we report genes such as *HRAS*, *MAPK3*, *MAP2K1*, *MAPK1*, *RPS6KB1* and *AKT1* in the leading edges for these gene set enrichments. Lastly, to our knowledge, enrichment of α6β4 integrin signaling (WikiPathways) in non-pure dHGP CRCLM has not been specifically described in previous literature. This signal is, in part, driven by all three subunits of laminin-332 (*LAMA3*, *LAMB3*, *LAMC2*), which has been described as marking the invasive tumor-stroma border of several epithelial cancers [28], as well as one of its integrin receptor subunits, *ITGB4*, which can produce downstream proliferative signaling in colon cancer [29] (Fig. 8e, f).

To summarize, by contrasting pure dHGP CRCLM with those that have a compromised or absent desmoplastic rim (i.e., non-pure dHGP) we corroborate several trends that have been reported to differentiate dHGP and rHGP more generally, e.g., accumulation of stromal cells and ECM reorganization in dHGP and increased glucose metabolism in others. Our findings add to previous characterizations of gene expression patterns of CRCLM HGPs by emphasizing an endothelial cell signal in pure dHGP, and laminin-integrin signaling in CRCLM with non-pure dHGP CRCLM.

### Weak association between high *CCR2* expression with better survival

The association between a compromised or absent desmoplastic rim in CRCLM and worse clinical outcomes is well-established [2, 3, 10, 11]. However, in our cohort, there remains unexplained variability in overall survival within both the dHGP and the rHGP groups. We hypothesized that the expression of certain genes that correlate with HGP group might have prognostic relevance beyond the histopathological classification of the MMPI.

Using single-gene expression levels as well as gene expression ratios derived from our list of differentially expressed genes, we built Cox regression models for overall survival after first metastasectomy including HGP group as a covariate.

First, we included all 52 patients with available survival data in this analysis and then repeated the analysis on the subset of 21 patients with pure or predominant dHGP CRCLM.

Although no significant results remained after correction for multiple testing, certain signals were nominally significant across all analyses. Specifically, high expression of the C–C chemokine receptor type 2 gene (*CCR2*)—and certain derivative ratios, for instance *CCR2* expression normalized by *ERBB2* expression—were nominally associated with better survival across all patients as well as across the patients in the subset of pure or predominant dHGP, independently of exact HGP types (supplementary fig. S5). Interestingly, these features tended to show stronger effect sizes within the subgroup of patients with pure or predominant dHGP.

## Discussion

In this study, we aimed to contribute to the molecular characterization of HGPs in CRCLM by using a commercially available targeted gene expression panel, contrasting pure dHGP against all other HGPs given its distinct clinical relevance, and including multiple samples per patient to assess the contribution of patient-specific factors to gene expression variability.

To this end, we analyzed gene expression profiles of CRCLM resection specimens using the NanoString nCounter® PanCancer Progression Panel, which can be easily implemented in routine laboratory workflows. By unsupervised clustering of the most variably expressed genes, we identified four gene clusters. Two of these showed significant enrichment for specific HGP types (pure dHGP in cluster 4 and pure rHGP in cluster 2), while pure dHGP was significantly depleted in cluster 1. These findings contrast with those of Latacz et al., who reported that clustering of gene expression data in CRCLM was driven primarily by factors unrelated to HGP [13]. A key methodological difference may explain this discrepancy: whereas Latacz et al. applied whole-transcriptome RNA sequencing, we used the NanoString nCounter® PanCancer Progression Panel, which targets a predefined set of genes involved in angiogenesis, EMT, ECM remodeling, and metastasis. This targeted approach likely increased the chance of detecting associations between gene expression variance and HGP type, which is discussed in more detail as a limitation at the end of the Discussion.

The most clinically relevant distinction is between pure dHGP and all other HGP types, as pure dHGP confers a significantly better prognosis in CRCLM [3, 10], a finding we reproduced in the survival analysis of the NanoString subcohort. We therefore performed DGEA comparing pure dHGP CRCLM to all non-pure dHGP CRCLM. This analysis revealed 25 genes significantly overexpressed in pure dHGP and 30 genes significantly overexpressed in non-pure dHGP CRCLM. To reveal the biological context of differentially expressed genes, we performed GSEA, identifying gene sets related to ECM organization, stromal and endothelial cell signals, and EMT enriched in pure dHGP, and glucose metabolism, α6β4 integrin signaling, and interleukin signaling enriched in non-pure dHGP.

Taken together, our GSEA findings largely recapitulate transcriptomic differences between dHGP and rHGP that have been reported in previous studies, including signals from stromal cells and fibroblasts, ECM reorganization, and EMT enriched in dHGP [12, 13], as well as increased glucose metabolism in rHGP metastases [11–13]. Our results are also in line with upregulation of inflammatory responses in dHGP as reported previously [12, 13]. However, this finding was not statistically significant in our data. The enrichment of interleukin signaling gene sets (i.e., IL6 and IL11 signaling pathways) in our cohort may seem contradictory in this regard, contrasting previous findings of upregulation of inflammatory and immune responses in dHGP [13]. The genes driving enrichment of these gene sets in our data, however, are associated with survival/proliferative signaling in general rather than being specifically linked to a cytokine response.

Notably, these established transcriptomic differences between dHGP and rHGP proved consistently re-identifiable in our data despite two key methodological differences from prior work: the use of a targeted, commercially available gene expression panel rather than whole-transcriptome sequencing, and a comparison strategy contrasting pure dHGP specifically against all other HGPs. This concordance suggests that the core biological differences distinguishing these growth patterns are robust across both analytical approaches and are captured within panel-based expression profiling.

Beyond corroborating these established patterns, our findings extend current understanding of HGP biology by identifying enrichment of α6β4 integrin signaling, driven by laminin-332 and *ITGB4*, as a feature of non-pure dHGP CRCLM that has not, to our knowledge, been previously described in this context. Within this pathway, the genes encoding the three subunits of the heterotrimer laminin-332 (*LAMA3*, *LAMB3,* and *LAMC2*) [30] were upregulated in non-pure dHGP CRCLM (although only *LAMB3* at *p*_adj_ < 0.05). Laminin-332 is known to promote tumor cell invasion and EMT [30]. In hepatocellular carcinoma, it acts synergistically with transforming growth factor-beta1 (TGFβ1) [31] and high expression of mRNA of its subunit Lamγ2 (*LAMC2*) correlates with the absence of complete encapsulation [32]. TGFβ1, in turn, has been implicated in promoting vessel co-option and thus the rHGP phenotype in CRCLM [33]. In colorectal adenocarcinoma, laminin-332 is considered a negative prognostic marker and has been linked to tumor cell budding and early liver metastases [34–36]. Other genes contributing to the enrichment of the α6β4 integrin signaling pathway belong to the PI3K–AKT–mTOR and MAPK/ERK pathways. However, none of these genes showed strong overexpression (i.e. fold change > 1.5) and most were below the level of significance of *p*_adj_< 0.05 in DGEA.

Consistent with the observations by Latacz et al. [13], HGP was not the dominant driver of gene expression variance in our cohort, being outweighed by patient- and tumor-specific factors (e.g., patient identity). To address intra-patient heterogeneity, we included multiple CRCLM per patient where available. Notably, patient identity itself emerged as the strongest non-HGP-related factor influencing gene expression. Patient identity was followed by mutation status, primary tumor grade, and HGP, which contributed to a similar extent. Other variables, such as gender, UICC stage, and primary tumor site, showed only minimal association with global gene expression variance. These findings indicate that patient- and tumor-specific factors substantially contribute to gene expression variability in CRCLM. In line with the findings by Latacz et al. [13], *RAS*/*BRAF* mutation status was not significantly different between HGPs, with only a non-significant trend toward enrichment of wild-type tumors in pure dHGP. As this study was not designed to identify a validated gene panel predictive of HGP, we did not formally assess the robustness of any HGP-associated set of genes against these patient- and tumor-specific factors.

However, with respect to these interpatient differences, we applied different strategies to reduce the effect of inter-patient heterogeneity on differential gene expression by either averaging the gene expression levels between samples with the same HGP per patient (strategy 1) or by reducing our analysis to only those patients with both a pure dHGP and a non-pure dHGP CRCLM (strategy 2). Given the limited power of strategy 2, we additionally considered effect-size concordance between strategies rather than relying on statistical significance alone and identified a set of genes consistently upregulated in pure dHGP across both strategies (especially *ITGA8*, *VCAM1*, and *CCR2*) and upregulated in non-pure dHGP across both strategies (*AGR2*, *SNRPF*, and *PTTG1*). Nonetheless, this concordance was limited to a small subset of the originally identified differentially expressed genes, and the remainder should be interpreted with appropriate caution given the constrained power of strategy 2 to independently confirm them.

Among these, *AGR2* stands out due to its consistent and statistically significant overexpression in non-pure dHGP CRCLM across both strategies. AGR2 (anterior gradient 2) is a protein disulfide isomerase (PDI) primarily involved in protein folding within the endoplasmic reticulum [37]. Beyond its intracellular role, AGR2 can be secreted into the extracellular tumor microenvironment, where it promotes invasiveness and metastatic potential [38]. AGR2 is overexpressed in a wide range of cancer types, and its overexpression is generally associated with poor prognosis [37]. In colorectal cancer, however, its biological role appears more complex. Several studies have reported that loss of AGR2 expression correlates with unfavorable outcomes [39, 40], a finding that may partly reflect its higher expression in tumors with high-grade microsatellite instability, which typically exhibit improved therapeutic responsiveness [40, 41]. Another study identified tumor-associated neutrophils as the predominant cell type for secreting AGR2 in colorectal cancer, promoting tumor cell migration and metastatic potential [42]. In this context, the consistent and significant overexpression of *AGR2* observed in non-pure dHGP CRCLM in our cohort is noteworthy, given that these metastases have significantly poorer prognosis than pure dHGP CRCLM, which exhibit markedly lower *AGR2* expression levels. These observations highlight the need for further investigation into the mechanistic role of *AGR2* in CRCLM, particularly in light of ongoing efforts to develop AGR2-targeted therapeutic strategies [37].

We also hypothesized that the expression of certain genes might correlate with survival independently of HGP. Although no associations in the Cox regression models remained significant after correction for multiple testing, high expression of *CCR2* showed a nominally significant association with improved survival, irrespective of HGP. Interestingly, *CCR2* is generally viewed as a tumorigenic factor by promoting metastasis in colorectal cancer [43, 44]. In this context, the finding that *CCR2* is a weakly positive prognostic factor independently of HGP in our cohort is intriguing. However, at the discovery stage, when correcting for the full search space of differentially expressed genes and gene-pair ratios, this association did not remain significant after multiple testing correction and should therefore be considered exploratory. Further studies are needed to determine whether *CCR2* expression provides prognostic information beyond that captured by HGP.

Several limitations of this study should be considered when interpreting the findings. It should be emphasized that the results of this study are exploratory in nature. Confirmation of individual biomarkers for clinical translation was not the intended objective. Publicly available RNA sequencing datasets of CRCLM remain scarce, and direct comparison with existing datasets is further limited by differences in tissue composition and in the classification and distribution of HGPs. Consequently, external validation of our results was not performed. Nevertheless, a detailed comparison of our findings with previously published data from comparable cohorts is provided in the Results and Discussion sections above. Patient identity was the dominant source of gene expression variance in our cohort, outranking HGP and other clinicopathological variables. While we identified a small set of genes with consistent directionality across independent analytical approaches, their robustness in predicting HGP independent of patient- and tumor-specific molecular background was not formally assessed in this study. Any future gene panel intended to predict HGP will need to demonstrate such robustness before translational application can be considered. Microdissection encompassed tumor tissue, the fibrotic rim (when present), and adjacent liver parenchyma. Consequently, the resulting gene expression profiles reflect a mixture of tumor-intrinsic and microenvironment-derived signals, precluding their separate analysis. This approach was intentionally chosen to capture the entire MMPI, as previous studies have demonstrated spatial heterogeneity in gene expression even within the desmoplastic rim itself. Another limitation of our approach is the use of a predefined gene panel instead of whole-transcriptome or total RNA sequencing. The PanCancer Progression Panel is enriched for pathways intrinsically linked to the dHGP and rHGP phenotypes themselves. This content-selection bias likely increases the sensitivity of our approach to detect HGP-associated differences in these specific pathways and should be considered when interpreting our findings, including the observation that unsupervised clustering with our targeted panel resolved HGP-associated clusters more clearly than in prior whole-transcriptome studies [13]. This difference may, at least in part, reflect panel composition rather than a true methodological advantage of targeted profiling over whole-transcriptome sequencing. We nevertheless chose this strategy deliberately, as targeted panels offer shorter turnaround times and greater feasibility for potential routine clinical implementation, and view our findings as complementary and hypothesis-generating rather than confirmatory of superiority over unbiased approaches. Finally, only a small proportion of patients in our retrospective cohort received anti-VEGF therapy prior to resection of CRCLM. Bevacizumab, an anti-VEGF antibody, was approved by the European Medicines Agency in 2005 following studies demonstrating a survival benefit when added to standard chemotherapy in metastatic colorectal cancer [45]. Anti-angiogenic treatment has been associated with HGP biology, particularly reduced response of vessel-co-opting rHGP metastases to this therapy and a potential shift toward rHGP CRCLM following anti-angiogenic treatment [46, 47]. However, the underrepresentation of patients treated with anti-VEGF systemic therapy in our cohort precluded a systematic assessment of its influence on gene expression patterns or survival.

We demonstrated that differential gene expression analysis revealed specific genes and pathways that differ between pure dHGP and non-pure dHGP CRCLM even though unsupervised gene expression clustering mainly did not align with HGP. While most of these differentially enriched pathways recapitulate findings from previous studies, our results corroborate a consistent biological signal distinguishing pure dHGP from non-pure dHGP CRCLM despite differences in cohort, methodology, and analytical approach. Beyond previous findings, our data suggest enrichment of α6β4 integrin signaling as a feature of non-pure dHGP CRCLM that has not, to our knowledge, been previously described in this context and may warrant further investigation.

## Supplementary Information

Below is the link to the electronic supplementary material.

Supplementary Material 1

## Data Availability

Raw gene expression data is deposited in the Gene Expression Omnibus (GEO) repository (GSE336139). All acquired data are stored locally at the Institute of Pathology of the University of Regensburg and will be provided upon reasonable request.
