# Supplementary material for: Molecular characterization of colorectal cancer liver metastases with pure desmoplastic versus non-pure desmoplastic histopathological growth patterns using a targeted gene expression panel
Source: Clin Exp Metastasis. 2026 Oct 12;43(5):54. doi: 10.1007/s10585-026-10433-x (PMC13654873; doi:10.1007/s10585-026-10433-x)
Supplement: Supplementary file 1 [file 10585_2026_10433_MOESM1_ESM.pdf]

Supplementary Materials

Journal: Clinical & Experimental Metastasis

Article Title: Molecular Characterization of Colorectal Cancer Liver Metastases with Pure Desmoplastic versus Non-Pure Desmoplastic Histopathological Growth Patterns Using a Targeted Gene Expression Panel

Felix Keil*, Andreas Lösch, Marco Mühlbauer, Tobias Pukrop, Raquel Blazquez, Hans Jürgen Schlitt, Karolina Müller, Maria Steiger, Yanren Linda Hu, Julia Maurer, Cathleen Brese, Michael Gerken, Matthias Evert, Daniela Hirsch, Rainer Spang, Stefan M. Brunner, Katja Evert

[*] corresponding author: Felix Keil; Institute of Pathology, University of Regensburg; 93053 Regensburg, Germany; felix1.keil@ur.de.

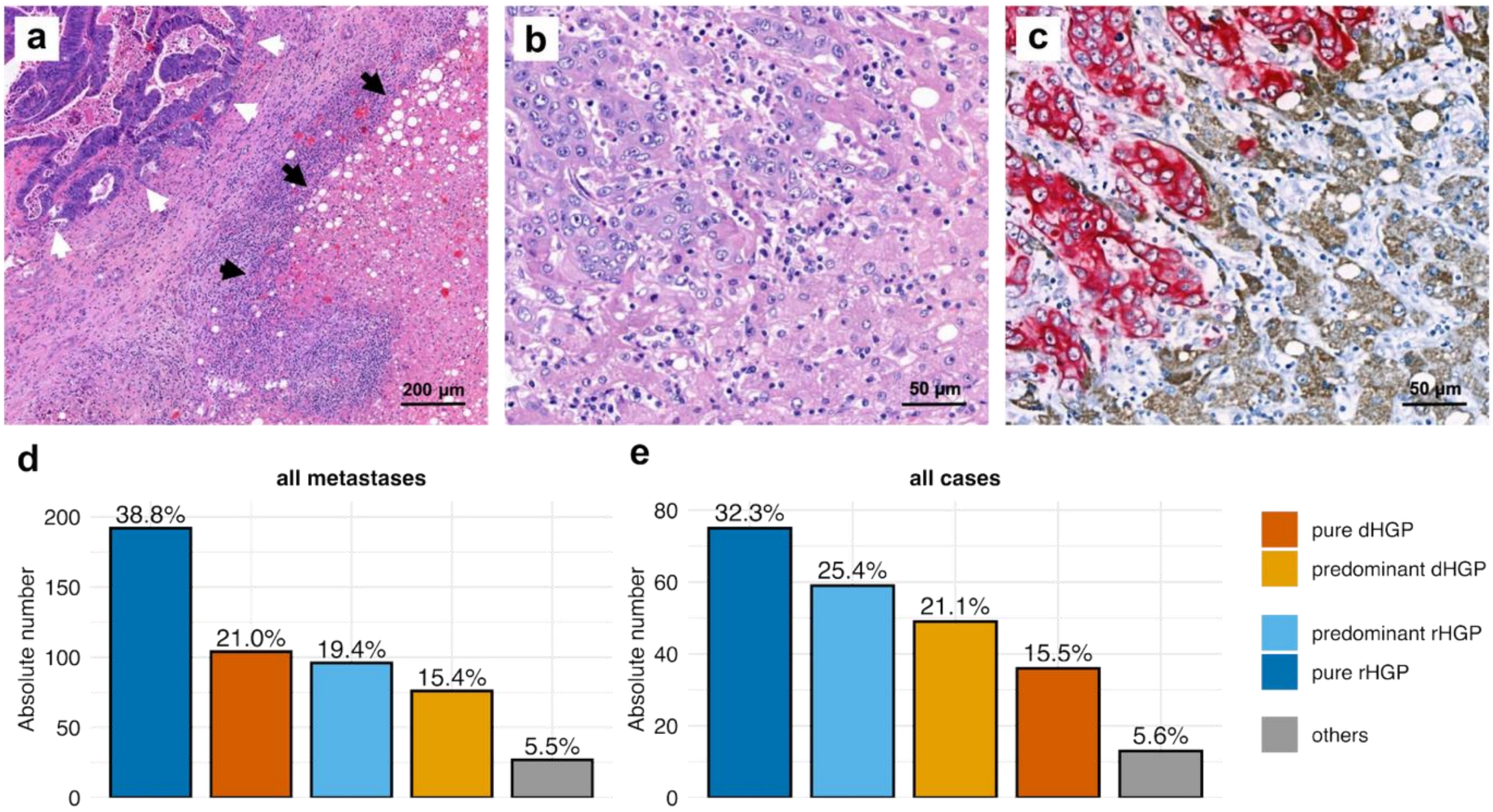

Fig. S1 Histomorphology of the most prevalent histopathological growth patterns (HGPs) of CRCLM and their distribution in the entire cohort.

(**a**) dHGP (HE, 50x): desmoplastic capsule separating metastasis (white arrowheads) from liver parenchyma (black arrowheads). (**b**) rHGP (HE, 200x): tumor cells (upper left) infiltrating liver parenchyma (lower right) by replacing hepatocyte trabeculae. (**c**) Same rHGP as in (b). CK20 (red) highlighting tumor cells, HepPar-1 (brown) highlighting hepatocytes. (**d**) Distribution of HGPs across all CRCLM (n=495). (**e**) Distribution of HGPs across all patients (n=232).

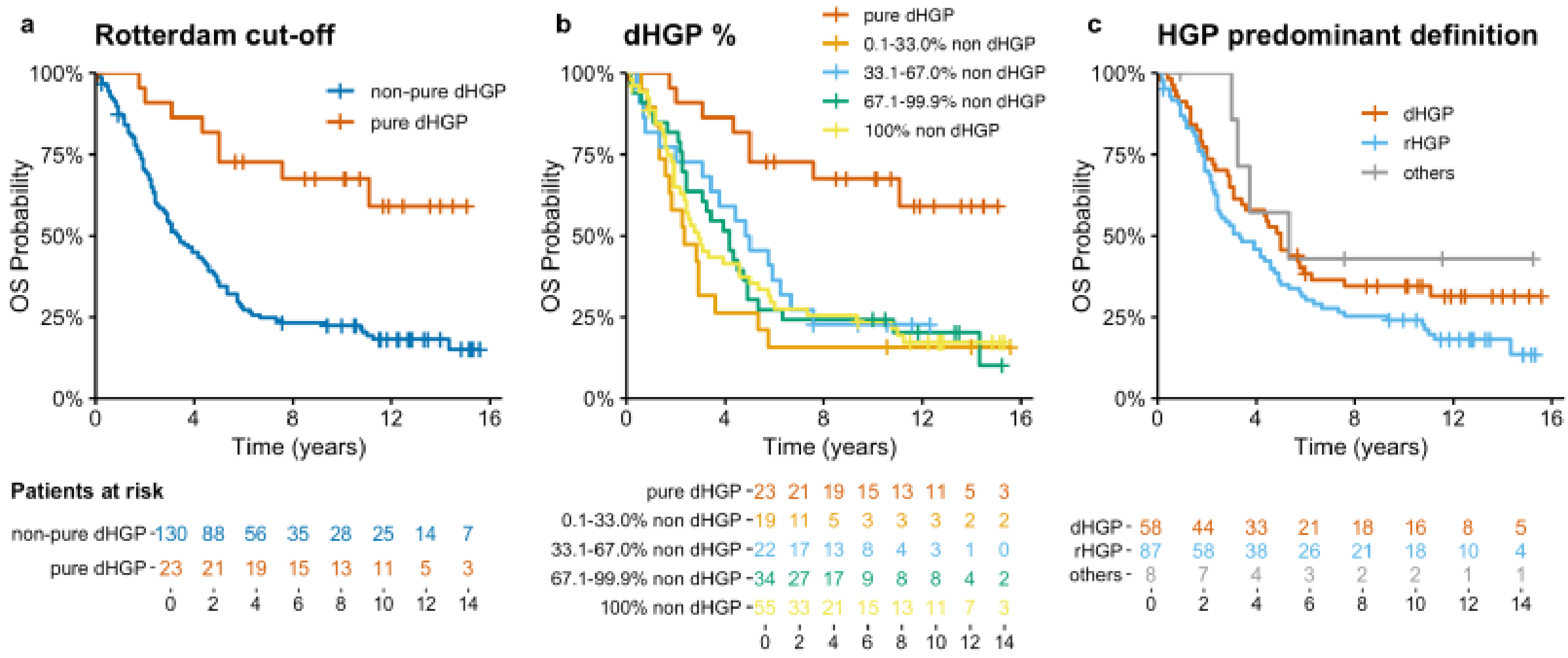

Fig. S2 Kaplan-Meier survival curves demonstrate longer overall survival in CRCLM with pure dHGP.

(**a**) Patients with pure dHGP (n=23) vs. patients with non-pure dHGP (n=130) ("Rotterdam cut-off") showing a significant difference in overall survival (OS) probability (p<0.001). (**b**) Patients stratified by extent of dHGP, also resulting in significant OS difference (p=0.002). (**c**) Patients stratified by predominant HGPs showing no significant difference in OS (p=0.249). All patterns other than dHGP or rHGP grouped as "other" due to small sample size. Estimated 2-year survival rates were 90.9% (95% CI 79.7-100.0%) for pure dHGP vs. 69.7% (95% CI 62.1-78.2%) for all non-pure dHGPs, and the 5-year survival rates were 72.7% (95% CI 56.3-93.9%) for pure dHGP vs. 34.4% (95% CI 27.1-43.9%) for non-pure dHGPs. Median overall survival (mOS) was not reached for pure dHGP. For all other patients combined, mOS was 40 months (95% CI: 30.5 - 49.5).

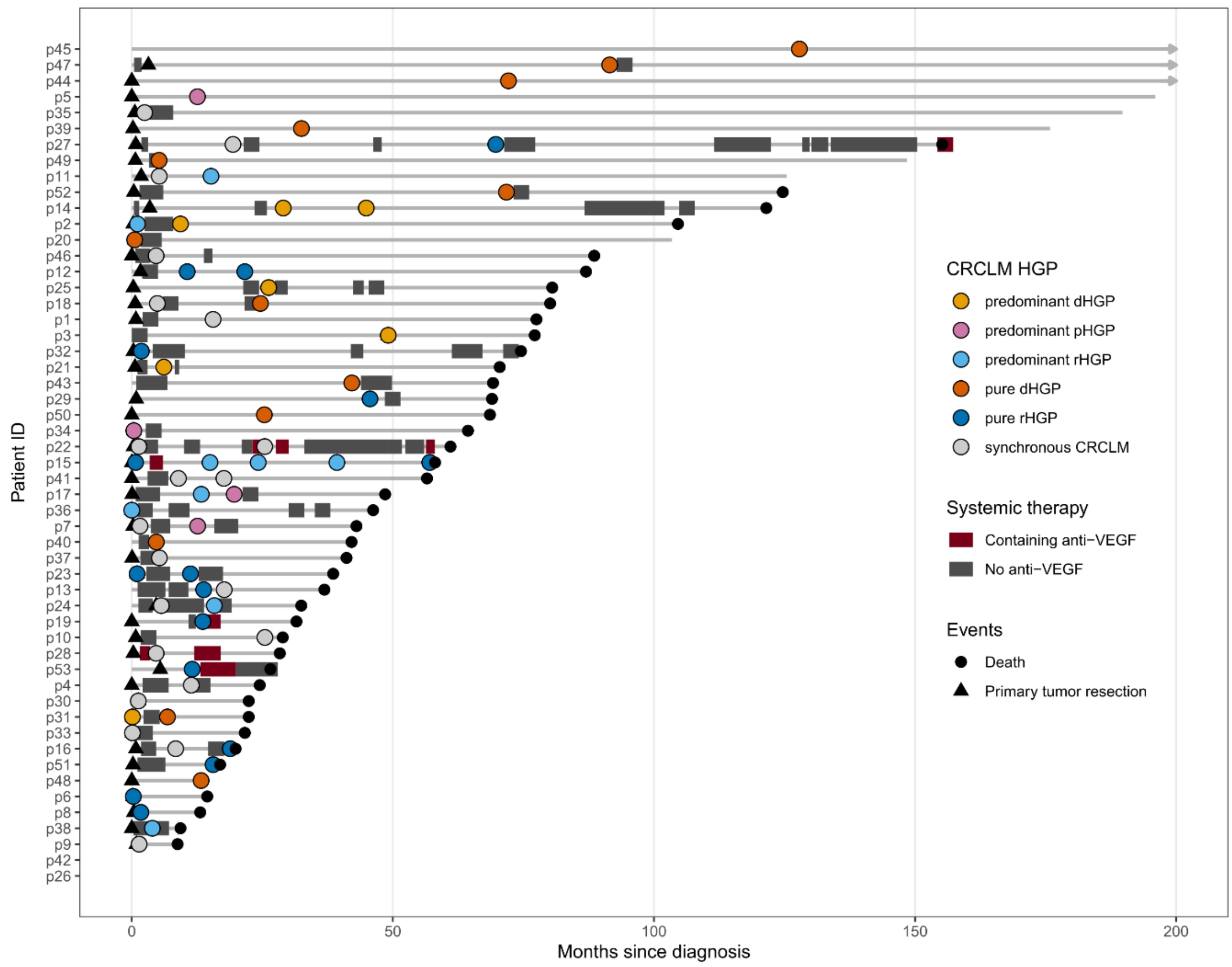

Fig. S3 Swimmer plot of patients with CRCLM analyzed by NanoString demonstrates heterogeneity in disease courses and previous systemic therapies.

Timelines are anchored to the date of primary diagnosis and display systemic therapies as horizontal bars; when treatment end dates were unknown, a fixed duration of 3 months was assumed. CRCLM analyzed by NanoString are shown as dots, with colors indicating HGP of individual metastases. For synchronous metastasectomies, dots are displayed in grey instead of an HGP-specific color, as potentially different HGPs may be present within a single surgical event. Patients 44, 45, and 47 had ongoing survival beyond 200 months of follow-up. Patients 26 and 42 are not shown due to unknown dates of primary diagnosis.

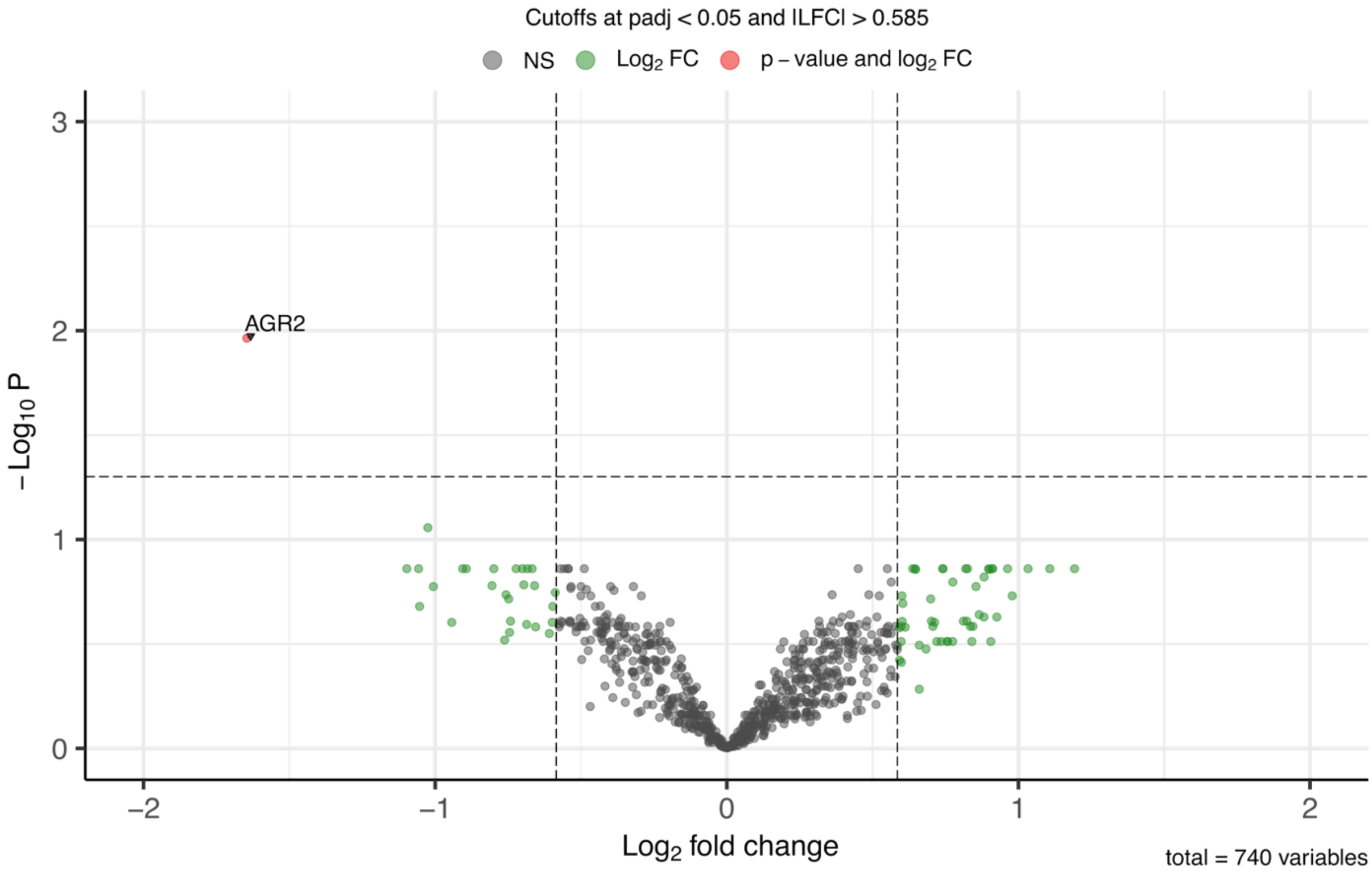

Fig. S4 *AGR2* remained significantly downregulated in pure dHGP CRCLM after restricting the analysis to only those patients that had at least one pure dHGP and one non-pure dHGP CRCLM (strategy 2).

Volcano plot shows that no significantly upregulated genes ($p_{adj} < 0.001$ and $\log_2$(fold change) > 0.585) in pure dHGP (upper right corner) remained after restricting the comparison to those 9 patients with at least one sample on each side of the comparison (pure dHGP versus non-pure dHGPs). LFC = $\log_2$(fold change)

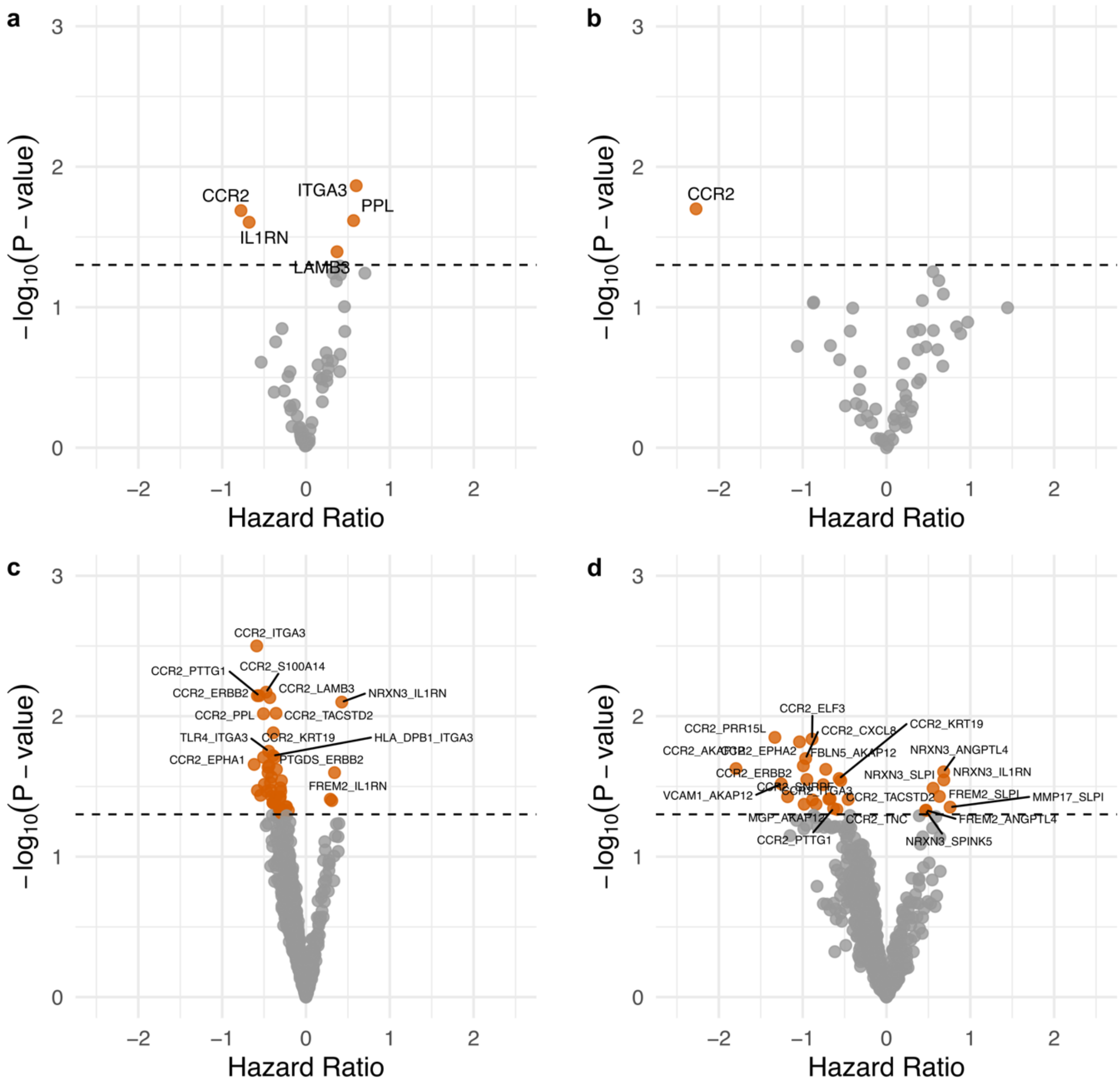

Fig. S5 Survival analysis on gene expression data highlights *CCR2* and derivative ratios as consistently associated with longer survival, however non-significant after correction for multiple testing.

(**a, b**) Volcano plots of Cox regression models including the 55 significantly and most differentially expressed genes between pure dHGP CRCLM and non-pure dHGP CRCLM including all patients (a) and only patients with pure or predominant dHGP CRCLM (b). (**c, d**) Ratios between the significantly upregulated genes in pure dHGP and non-pure dHGP CRCLM including all patients (c) and only patients with pure or predominant dHGP CRCLM (d). Orange dots significant at $p_{adj} < 0.05$.

Table S1 Genes encompassed by the NanoString nCounter® PanCancer Progression Panel. 740 target genes listed first, followed by the 30 internal reference genes.

AAMP	ABI3BP	ACHE	ACTG2	ACVR1	ACVR1C	ACVRL1	ADAM15	ADAM17
ADAM28	ADAM8	ADAM9	ADAMTS1	ADAMTS12	ADAMTS8	ADAP1	ADD1	ADM2
ADRA2B	AEBP1	AGGF1	AGR2	AGRN	AGT	AHNAK	AKAP12	AKAP2
AKT1	AKT2	AKT3	ALB	ALDOA	ALOX5	AMH	ANG	ANGPT1
ANGPT2	ANGPTL2	ANGPTL4	ANPEP	ANXA2P2	AP1M2	APC	APOD	APOE
APOH	AQP1	ARAP2	AREG	ARHGAP32	ARHGDIB	ASPN	ATPIF1	B3GNT3
BAD	BAG2	BAI1	BAI3	BCAS1	BGN	BICC1	BMP4	BMP5
BMP7	BMPER	BMPR1A	BMPR1B	BMPR2	BNC2	BRMS1	BTG1	C1S
C3	C3AR1	CADM1	CALCRL	CALD1	CAMK2A	CAMK2B	CAMK2D	CAMP
CASP8	CAV1	CBLC	CCBE1	CCDC80	CCL11	CCL21	CCL5	CCL7
CCL8	CCR2	CCR3	CD163	CD24	CD2AP	CD34	CD36	CD44
CD46	CD82	CDC42	CDH1	CDH11	CDH13	CDH2	CDK14	CDKN1A
CDKN2A	CDS1	CEACAM1	CEACAM5	CEACAM6	CEP170	CEP295	CFP	CGN
CHAD	CHD4	CHI3L1	CHP1	CHP2	CHRDL1	CHRNA7	CIB1	CKMT1A
CLDN1	CLDN3	CLDN4	CLDN7	CLEC2B	CLEC3B	CLIC4	CLU	CMA1
CNN1	COL18A1	COL1A1	COL1A2	COL3A1	COL4A1	COL4A2	COL4A6	COL5A1
COL5A2	COL6A1	COL6A2	COL6A3	COL7A1	COMP	CREBBP	CRIP2	CRISPLD2
CSF2RB	CSPG4	CST7	CTNNB1	CTNND1	CTSG	CTSH	CTSK	CTSL
CUL1	CX3CL1	CXADR	CXCL10	CXCL11	CXCL12	CXCL13	CXCL17	CXCL8
CXCR2	CXCR3	CXCR4	CYB561	CYBB	CYP1B1	DAG1	DCC	DCN
DDR2	DENND5A	DENR	DESI1	DICER1	DLC1	DLG1	DLL4	DPT
DPYSL3	DSC2	DST	ECM1	ECM2	ECSCR	EDN1	EGF	EGFL7
EGFR	EGLN2	EGLN3	EIF2AK3	EIF4E2	EIF4EBP1	ELF3	ELK3	EMCN
EMILIN1	EMILIN3	EMP3	ENO1	ENO2	ENO3	ENPEP	ENPP2	EP300
EPAS1	EPCAM	EPHA1	EPHA2	EPHB1	EPHB3	EPHB4	EPN3	EPS8L1
ERBB2	ERBB2IP	ERBB3	EREG	ERMP1	ESRP1	ETV4	EVI2A	EVPL
F11R	F3	FAM174B	FAP	FASLG	FBLN1	FBLN5	FBN1	FBN2
FBP1	FERMT2	FGF18	FGF2	FGF9	FGFR1	FGFR2	FGFR3	FGFR4
FGL2	FHL1	FIGF	FLI1	FLT1	FLT4	FMOD	FN1	FOXC2
FOXO4	FRAS1	FREM1	FREM2	FST	FSTL1	FUT3	FXYD6	GALNT7
GATA4	GDF15	GDF5	GDF6	GIMAP4	GIMAP6	GJA5	GLYR1	GPI
GPR124	GPR56	GPX1	GREM1	GRHL2	GSN	GTF2I	GZMK	HAPLN1
HAS1	HDAC5	HDHD3	HEG1	HGF	HIF1A	HIPK1	HIPK2	HK2
HK3	HKDC1	HLA-DPB1	HMOX1	HOXA5	HOXA7	HOXB13	HOXB3	HPSE

HRAS	HSD17B12	HSP90B1	HSPB1	HSPG2	HUNK	IBSP	ICAM1	ID1
ID2	ID4	IFNG	IGF1	IGFBP4	IGFBP7	IL10RA	IL11	IL13RA2
IL15	IL18	IL1A	IL1B	IL1RL1	IL1RN	IL6	ILK	INHBA
INHBE	IRF6	ISL1	ISLR	ITGA1	ITGA11	ITGA2	ITGA3	ITGA5
ITGA6	ITGA7	ITGA8	ITGA9	ITGAM	ITGB1	ITGB1BP1	ITGB2	ITGB3
ITGB4	ITGB6	ITGB7	ITGB8	ITM2A	JAG1	JAM2	JAM3	JUN
KCNJ8	KDM1A	KDR	KIAA1462	KISS1	KLK3	KRAS	KRIT1	KRT1
KRT14	KRT19	KRT7	LAD1	LAMA1	LAMA3	LAMA4	LAMA5	LAMB3
LAMC1	LAMC2	LDHA	LEFTY1	LGALS1	LHFP	LIFR	LLGL2	LOX
LOXL2	LRG1	LTBP4	LUM	LY96	MAF	MAP2K1	MAP2K2	MAP2K4
MAP3K7	MAPK1	MAPK3	MAPKAPK3	MCAM	MED1	MED23	MEG3	MEOX2
MET	MFAP4	MGAT5	MGP	MISP	MMP1	MMP10	MMP12	MMP13
MMP14	MMP17	MMP2	MMP24	MMP3	MMP9	MMRN2	MPDZ	MRC1
MS4A4A	MS4A6A	MT3	MTA1	MTBP	MTDH	MTOR	MUC1	MYC
MYCL	MYH11	MYLK	MYO1D	MYO5C	NAA15	NAP1L3	NCAM1	NCL
NDNF	NDP	NDRG1	NF1	NF2	NFAT5	NFATC2	NFKB1	NID2
NME1	NME4	NODAL	NOS2	NOS3	NOTCH1	NOX5	NPR1	NR3C1
NR4A1	NR4A3	NRCAM	NRP1	NRP2	NRXN1	NRXN3	NTRK1	OAS1
OCLN	OGN	OLFML2B	OVOL2	P3H1	P3H2	PCOLCE	PDCD10	PDCL3
PDGFA	PDGFC	PDGFRB	PDK1	PDPN	PEBP4	PECAM1	PFKFB1	PFKFB4
PGK1	PIK3CA	PIK3CD	PIK3CG	PIK3R1	PIK3R2	PIK3R5	PIK3R6	PITX2
PKM	PKN1	PKNOX1	PLA2G10	PLA2G2A	PLA2G2D	PLA2G3	PLAU	PLAUR
PLCG1	PLCG2	PLEKHO1	PLS1	PLXDC1	PLXNC1	PLXND1	PMP22	PNPLA6
POPDC3	POSTN	PPFIBP2	PPL	PPP1R16B	PPP2CB	PPP2R1A	PPP3R1	PRELP
PRF1	PRKCB	PRKCG	PRKCZ	PROK2	PROM1	PRR15L	PRSS22	PRSS8
PTEN	PTGDS	PTGIS	PTGS2	PTK2	PTK2B	PTK6	PTPRB	PTPRC
PTPRM	PTRF	PTTG1	PTX3	PXDN	PYCARD	QKI	RAB25	RAC1
RAC2	RAF1	RAMP1	RAMP2	RB1	RBL1	RBL2	RBM47	RBPJ
RBX1	RELN	RGCC	RHOA	RNH1	ROBO4	ROCK1	ROCK2	RORA
RORB	RPS27A	RPS6KB1	RPS6KB2	RRAS	RTN4	RUNX1	RUNX1T1	S100A14
S100A7	S1PR1	SACS	SAMSN1	SCG2	SCNN1A	SDC4	SELE	SEMA3E
SERINC5	SERPINA1	SERPINE1	SERPINF1	SERPING1	SERPINH1	SET	SETD2	SFRP1
SFRP2	SH2B3	SH2D3A	SH3YL1	SHB	SIRT1	SKP1	SLC12A6	SLC2A1
SLC35A3	SLC37A1	SLC44A4	SLIT2	SLPI	SMAD1	SMAD2	SMAD3	SMAD4
SMAD5	SMAD9	SMC3	SMOC1	SMURF1	SMURF2	SNAI1	SNAI2	SNAI3
SNRPF	SOD1	SORD	SOX17	SOX2	SOX9	SP1	SPARC	SPARCL1
SPDEF	SPHK2	SPINK5	SPINT1	SPOCK3	SPP1	SRC	SRF	SRGN

SRPK2	SRPX2	SSTR2	ST14	STAB1	STAB2	STAT1	STAT3	SULF1
SV2B	SYK	SYNE1	TACSTD2	TAL1	TBX1	TBX4	TBXA2R	TCEB1
TCEB2	TCF20	TCF3	TCF4	TDGF1	TEK	TF	TFDP1	TFPI2
TGFB1	TGFB2	TGFBI	TGFBR2	THBS1	THBS2	THBS4	THY1	TIE1
TIMP1	TIMP2	TIMP4	TJP2	TJP3	TLR4	TMC6	TMEM100	TMEM30B
TMPRSS2	TMPRSS4	TMPRSS6	TNC	TNF	TNFRSF12A	TNFRSF1A	TNFSF10	TNFSF12
TNFSF13	TNMD	TNN	TNS1	TNXB	TOM1L1	TP53	TPM2	TPSB2
TPSD1	TSHR	TSPAN1	TWIST1	TWIST2	TXNIP	TYMP	UBA52	UTS2
VAMP8	VASH1	VAV2	VAV3	VCAM1	VCAN	VEGFA	VEGFB	VEGFC
VEZF1	VHL	VIM	VIT	VPS13A	VSIG4	VWA1	VWA2	WARS
WIPF1	WNT5A	WNT5B	WWTR1	ZC3H12A	ZCCHC24	ZEB1	ZEB2	ZFPM2
ZFYVE16	ZFYVE9							
**Internal Reference Genes**								
AGK	AMMECR1L	CC2D1B	CNOT10	CNOT4	COG7	DDX50	DHX16	DNAJC14
EDC3	EIF2B4	ERCC3	FCF1	GPATCH3	HDAC3	MRPS5	MTMR14	NOL7
NUBP1	PRPF38A	SAP130	SF3A3	TLK2	TMUB2	TRIM39	USP39	ZC3H14
ZKSCAN5	ZNF143	ZNF346						

Table S2 Cohort characteristics.

**Number of patients**	n = 232
**Sex**	
Female	82 (35.3%)
Male	150 (64.7%)
**Median age at primary diagnosis (years)**	61 (IQR 52-66)
**Primary tumor site**	
Cecum and ascending colon	28 (12.1%)
Right colonic flexure to sigmoid colon	103 (44.4%)
Rectum	78 (33.6%)
N/A	23 (9.9%)
**Primary tumor grade**	
G1	2 (0.9%)
G2	159 (68.5%)
G3	46 (19.8%)
N/A	25 (10.7%)
**UICC stage at primary diagnosis**	
Stage I	13 (5.6%)
Stage II	23 (9.9%)
Stage III	40 (17.2%)
Stage IV	114 (49.1%)
N/A	42 (18.1%)
**Number of metastases**	n = 495
Metastases per patient (median)	1.5 (IQR 1-3, range 1-14)
Patients with synchronous metastases	61/232 (26.3%)
Patients with metachronous metastases	39/232 (16.8%)

Table S3 Genes consistently upregulated in pure dHGP across both differential expression analyses (strategies 1 and 2). Genes exceeding the effect size threshold (fold change (FC) ≥150%) with a consistent direction of effect in both analyses are ranked by the rank product of the Wald statistic from both analyses.

gene	p_{adj} strategy 1	p_{adj} strategy 2	$log_2(FC)$ strategy 1	$log_2(FC)$ strategy 2	Wald statistic strategy 1	Wald statistic strategy 2	Wald rank strategy 1	Wald rank strategy 2	Wald rank product
ITGA8	0.009201668	0.137932662	0.741179871	0.825625804	3.502980346	2.764445093	721	726	723.4956807
VCAM1	0.005976506	0.202176018	0.59784481	0.603502518	3.69958437	2.393997531	727	713	719.9659714
CCR2	0.004663648	0.246505589	0.677206347	0.601198356	3.790567332	2.170349102	728	700	713.8627319
BAI3	0.013421564	0.15112442	0.643756304	0.882259215	3.235006787	2.699539347	704	723	713.4367526
PTGDS	0.026464543	0.245798517	0.808111443	0.811274121	2.857961202	2.202774479	671	703	686.8136574
OGN	0.00010764	0.323357861	1.751044788	0.585630105	5.129237539	1.65306189	740	634	684.9525531
SELE	0.01043754	0.307566559	0.924943759	0.75733959	3.423629894	1.739719855	719	649	683.1039452
PRELP	0.013421564	0.307566559	0.728129316	0.597723488	3.231057121	1.776626354	702	662	681.7066818
SEMA3E	0.054536253	0.137932662	0.617432215	1.033055629	2.492744739	2.804648759	638	728	681.5159573
FREM2	0.021772626	0.307566559	1.111409629	0.720139455	2.983042961	1.756981067	683	657	669.8738687
MFAP4	0.06273913	0.262872762	0.597814631	0.611692042	2.410306354	1.980910744	631	683	656.4853388

Table S4 Genes consistently upregulated in non-pure dHGP across both differential expression analyses (strategies 1 and 2). Genes exceeding the effect size threshold (fold change (FC) ≥150%) with a consistent direction of effect in both analyses are ranked by the rank product of the Wald statistic from both analyses.

gene	p_{adj} strategy 1	p_{adj} strategy 2	log_2(FC) strategy 1	log_2(FC) strategy 2	Wald statistic strategy 1	Wald statistic strategy 2	Wald rank strategy 1	Wald rank strategy 2	Wald rank product
AGR2	0.00010764	0.010865404	-1.864945262	-1.645995491	-5.227052578	-4.333391311	1	1	1
SNRPF	0.00205508	0.137932662	-0.686516182	-0.798985799	-4.055681009	-3.335095753	9	3	5.196152423
PTTG1	0.002678562	0.137932662	-0.854964832	-0.905843715	-3.968278906	-3.109542616	10	6	7.745966692
S100A14	0.015487018	0.137932662	-0.810049009	-0.893499045	-3.173319029	-2.908422032	30	9	16.43167673
KRT19	0.001979284	0.249134351	-1.043616317	-0.598715396	-4.106783897	-2.159329837	7	50	18.70828693
PLA2G2A	0.00205508	0.249134351	-1.881690111	-0.943308601	-4.068019614	-2.136944601	8	53	20.59126028
CEACAM6	0.017871765	0.137932662	-1.167269041	-1.056883569	-3.0860002	-2.760106045	32	16	22.627417
PRR15L	0.023268772	0.166274565	-0.638287885	-0.659274869	-2.938161477	-2.621159743	43	19	28.58321186
TSPAN1	0.009795055	0.245798517	-1.064835321	-0.741682439	-3.459530958	-2.217832681	20	43	29.3257566
BMP7	0.094682908	0.208925823	-0.837472576	-1.053432273	-2.169190543	-2.358446748	95	37	59.28743543
CKMT1A	0.052750697	0.281580918	-0.655862125	-0.608727507	-2.511917804	-1.867518944	66	90	77.07139547
